# The Bohr Effect Is Not a Likely Promoter of Renal Preglomerular Oxygen Shunting

**DOI:** 10.3389/fphys.2016.00482

**Published:** 2016-10-27

**Authors:** Ufuk Olgac, Vartan Kurtcuoglu

**Affiliations:** ^1^The Interface Group, Institute of Physiology, University of ZurichZurich, Switzerland; ^2^National Center of Competence in Research, Kidney.CHZurich, Switzerland; ^3^Zurich Center for Integrative Human Physiology, University of ZurichZurich, Switzerland

**Keywords:** renal oxygenation, oxygen shunting, carbon dioxide shunting, pH, Bohr effect

## Abstract

The aim of this study was to evaluate whether possible preglomerular arterial-to-venous oxygen shunting is affected by the interaction between renal preglomerular carbon dioxide and oxygen transport. We hypothesized that a reverse (venous-to-arterial) shunting of carbon dioxide will increase partial pressure of carbon dioxide and decrease pH in the arteries and thereby lead to increased oxygen offloading and consequent oxygen shunting. To test this hypothesis, we employed a segment-wise three-dimensional computational model of coupled renal oxygen and carbon dioxide transport, wherein coupling is achieved by shifting the oxygen-hemoglobin dissociation curve in dependence of local changes in partial pressure of carbon dioxide and pH. The model suggests that primarily due to the high buffering capacity of blood, there is only marginally increased acidity in the preglomerular vasculature compared to systemic arterial blood caused by carbon dioxide shunting. Furthermore, effects of carbon dioxide transport do not promote but rather impair preglomerular oxygen shunting, as the increase in acidity is higher in the veins compared to that in the arteries. We conclude that while substantial arterial-to-venous oxygen shunting might take place in the postglomerular vasculature, the net amount of oxygen shunted at the preglomerular vasculature appears to be marginal.

## Introduction

Hypoxic conditions in the renal cortex or in the medulla may induce tissue damage and contribute to the pathogenesis of acute and chronic kidney diseases (Evans et al., 2008). It is therefore essential to understand the mechanisms that are involved in the regulation of renal oxygenation. We have shown previously that preglomerular arterial-to-venous (AV) oxygen shunting—a mechanism hypothesized to contribute to the regulation of renal oxygenation (Leong et al., 2007; Evans et al., 2008)—is unlikely to be significant (Olgac and Kurtcuoglu, 2015a,b). Since then the question has arisen whether substantial shunting may nevertheless exist, enabled by the influence of preglomerular carbon dioxide dynamics on oxygen transport. Here we hypothesize that reverse, venous-to-arterial (VA) shunting of carbon dioxide will increase arterial partial pressure of carbon dioxide, decrease pH, and thereby lead to augmented oxygen offloading through the Bohr effect (Kilmartin and Rossi-Bernardi, 1973; Dash et al., 2016) and consequent AV oxygen shunting.

Measurements of renal carbon dioxide partial pressure (P_CO_2__) and pH indicate a wide range of values in various structures of the kidney: In the proximal tubule, P_CO_2__ was measured by Sohtell (1979) to be 60.6 mmHg, by DuBose et al. (1979) 65 mmHg, by Maddox et al. (1984) 57.1–62.1 mmHg, and by De Mello Aires et al. (1990) 35.5 mmHg. In stellate vessels, reported values are 33.7 mmHg (Sohtell, 1979), 65 mmHg (DuBose et al., 1979), 57.1 mmHg (Maddox et al., 1984), and 38.9 mmHg (De Mello Aires et al., 1990). pH of 6.7–7.06 was measured by DuBose et al. (1979) in the proximal tubule and of 7.27 in the stellate vessels. These values suggest that, depending on the location within the renal cortex, P_CO_2__ and pH may vary. However, the extent of the deviation of P_CO_2__ and pH from the values of systemic arterial blood is not well known. Similarly, reliable quantitative values of *in vivo* P_CO_2__ and pH values inside red blood cells (RBCs) traversing the preglomerular vasculature are, to our knowledge, not obtainable experimentally. Since these data are necessary to test our hypothesis, a mathematical modeling approach is warranted.

Renal CO_2_ trapping through a reverse shunting mechanism was previously hypothesized by Bidani et al. (1984) to occur in the postglomerular vasculature and by Atherton et al. (1988) and Schurek et al. (1990) in the interlobular artery-vein pairs of the preglomerular vasculature. Schurek et al. (1990) further hypothesized that such a mechanism enhances AV oxygen shunting through the Bohr effect. Here we tested this hypothesis incorporating the latest structural data by Ngo et al. (2014) that account for the wrapping of veins around arteries in the preglomerular vasculature. To this end, we extended our previous computational model on renal oxygen transport (Olgac and Kurtcuoglu, 2015a,b) to include carbon dioxide transport. We then coupled oxygen transport to carbon dioxide transport through a function describing the shift of the oxygen-hemoglobin dissociation curve with respect to the local P_CO_2__ and pH as recently described by Dash et al. (2016). The resulting computational model is capable of quantifying P_O_2__, P_CO_2__ and pH as well as fluxes of O_2_ and CO_2_ through the vessel walls of wrapped and not-wrapped artery-vein pairs in the preglomerular vasculature. Computations of oxygen transport were performed with a Hill equation with constant P_50_ as well as a variable P_50_ in dependence of the local P_CO_2__ and pH.

## Methods

### Model domain

The three-dimensional (3D) model domain was previously described in Olgac and Kurtcuoglu (2015a,b). It consists of representative levels corresponding to 11 Strahler orders of the preglomerular vasculature. Each level contains wrapped and not-wrapped artery-vein pairs. The structural information for each level (artery radius, R_a_, vein radius, R_v_, number of wrapped and not-wrapped vessels, k_w_ and k_nw_, respectively, lumen separation for wrapped and not-wrapped vessels, LS_w_ and LS_nw_, respectively, and length of vessels, l) is given in Table 1.

**Table 1:** **Structural information at representative levels**.

**Level**	**R_v_, μm**	**R_a_, μm**	**k_nw_**	**k_w_**	**l, mm**	**LS_nw_, μm**	**LS_w_, μm**	**φ**
0	10.79	7.66	58,348	10,216	0.155	152.1	11.2	0.0518
1	14.72	10.2	17,755	12,804	0.248	112.7	10.6	0.0238
2	26.16	17	5379	3879	0.315	112.7	10.6	0.0115
3	40.13	24.5	1700	1226	0.625	112.7	10.6	0.0115
4	50.30	29.7	288	922	0.82	86.1	11.7	0.0123
5	69.22	38.8	99	319	1.05	86.1	11.7	0.0123
6	114.07	59.3	18	121	1.15	86.6	17.9	0.0099
7	177.04	85	5	33	1.7	86.6	17.9	0.0099
8	285.63	126	1	8	6.13	86.6	17.9	0.0055
9	428.05	171	1	3	3.09	86.6	17.9	0.0055
10	603.77	223	1	1	3.12	86.6	37.0	0.0055

*R_v_, vein radius; R_a_, artery radius; k, no. of vessels; l, length of vessels; LS, lumen separation; φ, fractional capillary volume; nw, not-wrapped vessels; w, wrapped vessels*.

### Mathematical formulation

Two different sets of equations governing oxygen and carbon dioxide transport dynamics in the renal cortex are solved in the vessels and the tissue. The vessels are composed of a RBC-rich region in the core and a RBC-free region close to the walls. The thickness of the RBC-free region in vessels of each Strahler order is given by $\delta \;=\text{ R }+\;{\text{t}}_{\text{RBC}}\;-\;\sqrt{{\text{R}}^{2}\;-\;{\text{R}}_{\text{RBC}}^{2}}$, where R is the vessel radius in that order, and R_RBC_ and t_RBC_ are the radius (4 μm) and maximum half thickness (1.3 μm) of a RBC, respectively (Nair et al., 1989). The plasma and RBC velocity profiles and the hematocrit profile in the RBC-rich region, u_P_(r), u_RBC_(r) and h(r), respectively, as well as the plasma velocity profile in the RBC-free region, ${\text{u}}_{\text{p}}^{\prime }$ (r), are calculated as explained in Olgac and Kurtcuoglu (2015a) and set on the computational domain prior to solving the governing equations for oxygen and carbon dioxide transport.

#### Oxygen transport

For the oxygen transport, we base our calculations on a derivative of our model presented in Olgac and Kurtcuoglu (2015a) where now the variability of P_50_, i.e., the half saturation oxygen partial pressure, is taken into account. Briefly, in the vessels, blood plasma, and RBCs are axially convected. The plasma carries dissolved oxygen and the RBCs carry dissolved and hemoglobin-bound oxygen. Radial diffusion of dissolved oxygen in the plasma is also accounted for. For a variable P_50_, in the RBC-rich region, P_O_2__ is governed by

(1) $$\begin{array}{l} \left[\alpha_{{\text{O}}_{2}}^{\text{P}}\left(1-\text{h}\left(\text{r}\right)\right){\text{u}}_{\text{P}}\left(\text{r}\right)\;+\;\alpha_{{\text{O}}_{2}}^{\text{RBC}}\text{h}\left(\text{r}\right){\text{u}}_{\text{RBC}}\left(\text{r}\right)\left(\text{​}1\;+\;\frac{{\text{C}}_{\text{HbT}}}{\alpha_{{\text{O}}_{2}}^{\text{RBC}}}\;\frac{{\text{dS}}_{{\text{O}}_{2}}}{{\text{dP}}_{{\text{O}}_{2}}}\text{​}\right)\text{​​}\right]\\ \;\frac{\partial {\text{P}}_{{\text{O}}_{2}}}{\partial \text{z}}-\text{h}\left(\text{r}\right){\text{u}}_{\text{RBC}}\left(\text{r}\right){\text{C}}_{\text{HbT}}\frac{{\text{P}}_{{\text{O}}_{2}}}{{\text{P}}_{50}}\;\frac{{\text{dS}}_{{\text{O}}_{2}}}{{\text{dP}}_{{\text{O}}_{2}}}\;\frac{\partial {\text{P}}_{50}}{\partial \text{z}}\\ \;=\frac{\alpha_{{\text{O}}_{2}}^{\text{P}}{\text{D}}_{{\text{O}}_{2}}^{\text{P}}}{\text{r}}\frac{\partial }{\partial \text{r}}\left(\text{r }\frac{\partial {\text{P}}_{{\text{O}}_{2}}}{\partial \text{r}}\right), \end{array}$$

where C_HbT_, $\alpha_{{\text{O}}_{2}}^{\text{P}}$ and $\alpha_{{\text{O}}_{2}}^{\text{RBC}}$ are the total heme group concentration in RBCs and the solubility of oxygen in plasma and inside the RBC, respectively (see Data Sheet 1 in Supplementary Material for derivation). The permeability of oxygen in plasma is defined as ${\text{K}}_{{\text{O}}_{2}}^{\text{P}}=\alpha_{{\text{O}}_{2}}^{\text{P}}{\text{D}}_{{\text{O}}_{2}}^{\text{P}}$, where ${\text{D}}_{{\text{O}}_{2}}^{\text{P}}$ is the diffusion coefficient of oxygen in plasma. Note that the second term on the left hand side of Equation 1 vanishes for constant P_50_, recovering the original equation developed in Olgac and Kurtcuoglu (2015a). In the RBC-free region, plasma free oxygen concentration follows:

(2) $$\left[\alpha_{\text{P}}{\text{u}}_{\text{P}}^{\prime }(\text{r})\right]\frac{\partial {\text{P}}_{{\text{O}}_{2}}}{\partial \text{z}}=\frac{\alpha_{{\text{O}}_{2}}^{\text{P}}{\text{D}}_{{\text{O}}_{2}}^{\text{P}}}{\text{r}}\frac{\partial }{\partial \text{r}}\left(\text{r}\frac{\partial {\text{P}}_{{\text{O}}_{2}}}{\partial \text{r}}\right).$$

The tissue, together with its capillary vessels, is considered a homogeneous structure in which perfusion and consumption are uniform and dependent on the fractional capillary volume, φ. Both diffusion of free oxygen and advection of free and hemoglobin-bound oxygen along the capillaries are taken into account. The homogeneous tissue oxygen partial pressure, P_O_2__, is governed by Salathe (1982) and Olgac and Kurtcuoglu (2015a):

(3) $$\begin{array}{l} \varphi \text{u}\alpha_{\text{T}}\nabla {\text{P}}_{{\text{O}}_{2}}\left(1+\frac{{\text{H}}_{\text{c}}{\text{C}}_{\text{HbT}}}{\alpha_{{\text{O}}_{2}}^{\text{T}}}\frac{{\text{dS}}_{{\text{O}}_{2}}}{{\text{dP}}_{{\text{O}}_{2}}}\right)=\alpha_{{\text{O}}_{2}}^{\text{T}}{\text{D}}_{{\text{O}}_{2}}^{\text{T}}\nabla^{2}{\text{P}}_{{\text{O}}_{2}}\\ \;+\left(1-\varphi \right)({\dot{\text{M}}}_{{\text{O}}_{2}}+{\dot{\text{M}}}_{\text{c},{\text{O}}_{2}}), \end{array}$$

where u, $\alpha_{{\text{O}}_{2}}^{\text{T}}$, H_c_, ${\dot{\text{M}}}_{{\text{O}}_{2}}$ and ${\dot{\text{M}}}_{\text{c},{\text{O}}_{2}}$ are the advection velocity of oxygen in capillaries, solubility of oxygen in the tissue, hematocrit in the capillaries, oxygen consumption rate in the tissue and capillary source/sink term, respectively. The permeability of oxygen in tissue is defined such that ${\text{K}}_{{\text{O}}_{2}}^{\text{T}}=\alpha_{{\text{O}}_{2}}^{\text{T}}{\text{D}}_{{\text{O}}_{2}}^{\text{T}}$, where ${\text{D}}_{{\text{O}}_{2}}^{\text{T}}$ is the diffusion coefficient of oxygen in tissue. Details on how the capillary source/sink term is treated are given in Olgac and Kurtcuoglu (2015a). Advection of oxygen in capillaries is only considered in the tissue between the not-wrapped pairs, since the tissue between the wrapped artery-vein pairs is free of capillaries (Ngo et al., 2014).

In Equations (1–3), S_O_2__ is the saturation of hemoglobin with oxygen, which is represented by the Hill equation (Clark et al., 1985):

(4) $${\text{S}}_{{\text{O}}_{2}}=\frac{{\left({\text{P}}_{{\text{O}}_{2}}/{\text{P}}_{50}\right)}^{\text{n}}}{1\;+\;{\left({\text{P}}_{{\text{O}}_{2}}/{\text{P}}_{50}\right)}^{\text{n}}},$$

where n is an empirical constant. Its derivative with respect to P_O_2__ in Equations (1–3) is given by Olgac and Kurtcuoglu (2015a):

(5) $$\frac{{\text{dS}}_{{\text{O}}_{2}}}{{\text{dP}}_{{\text{O}}_{2}}}=\frac{\text{n}{\left({\text{P}}_{{\text{O}}_{2}}/{\text{P}}_{50}\right)}^{\text{n}}}{{\text{P}}_{{\text{O}}_{2}}{\left(1+{\left({\text{P}}_{{\text{O}}_{2}}/{\text{P}}_{50}\right)}^{\text{n}}\right)}^{2}}.$$

**Figure 1:**
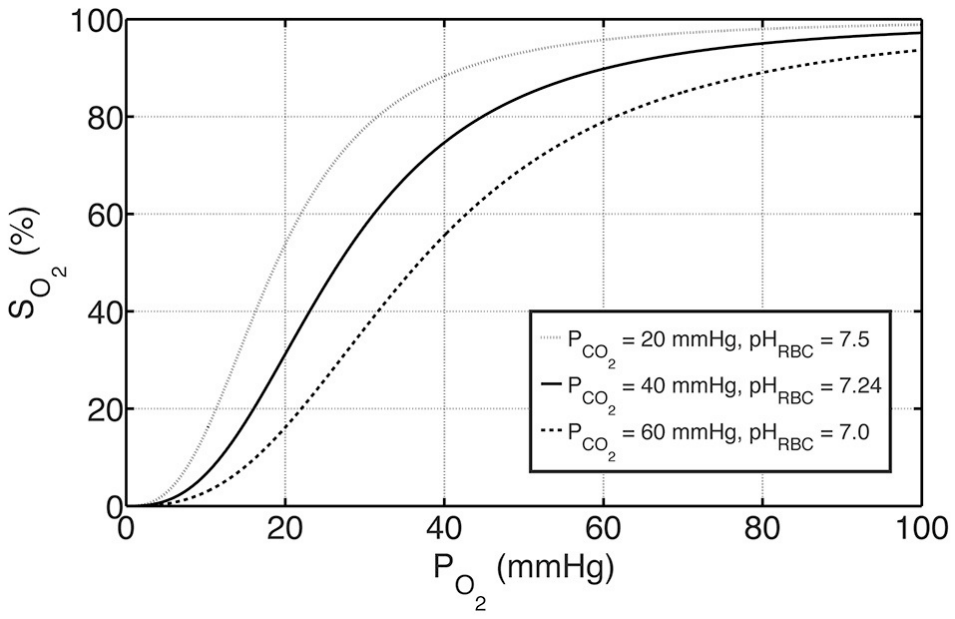
**The effects of P_**CO**_**2**__ and pH on oxygen-hemoglobin dissociation curve represented by S_**O**_**2**__ according to Equations (4) and (6)**. The solid curve in the middle represents standard physiological conditions with P_CO_2__ = 40 mmHg, pH_RBC_ = 7.24 and P_50_ = 26.8 mmHg, whereas the curves above and below represent examples of basic and acidic conditions, respectively. For these conditions, P_50_ is altered with respect to P_CO_2__ and pH by Equation (6a–c).

Equations (1–3) are solved either with a constant P_50_ (as was done in (Olgac and Kurtcuoglu, 2015a)) or with a variable P_50_ that is dependent on local P_CO_2__ and pH. P_50_ is varied with respect to local P_CO_2__ and pH according to Dash et al. (2016):

(6a) $$\begin{array}{l} {\text{P}}_{50,\Delta \text{pH}}\;=\;{\text{P}}_{50,\text{S}}\;-\;25.535\left(\text{pH }-\;{\text{pH}}_{\text{S}}\right)\;+\;10.646{\left(\text{pH }-\;{\text{pH}}_{\text{S}}\right)}^{2}\\ -\;1.764{\left(\text{pH }-\;{\text{pH}}_{\text{S}}\right)}^{3}, \end{array}$$

(6b) $$\begin{array}{l} {\text{P}}_{50,\Delta {\text{CO}}_{2}}\;=\;{\text{P}}_{50,\text{S}}\;+\;0.1273\left({\text{P}}_{{\text{CO}}_{2}}\;-\;{\text{P}}_{{\text{CO}}_{2},\text{S}}\right)\\ \;+\;1.083\cdot 10^{\;-\;4}{\left({\text{P}}_{{\text{CO}}_{2}}\;-\;{\text{P}}_{{\text{CO}}_{2},\text{S}}\right)}^{2}, \end{array}$$

(6c) $${\text{P}}_{50}={\text{P}}_{50,\text{S}}\left(\frac{{\text{P}}_{50,\Delta \text{pH}}}{{\text{P}}_{50,\text{S}}}\right)\left(\frac{{\text{P}}_{50,\Delta {\text{CO}}_{2}}}{{\text{P}}_{50,\text{S}}}\right),$$

where P_50, ΔpH_ and P_50, ΔCO_2__ represent the shifts in P_50_ with respect to pH and P_CO_2__, respectively, and standard physiological values are denoted with “S,” for which P_50, S_ = 26.8 mmHg, pH_S_ = 7.24 and P_CO_2_, S_ = 40 mmHg (Dash et al., 2016). Oxygen-hemoglobin dissociation curves under these physiological reference conditions as well as under exemplary acidic and basic conditions are shown in Figure 1.

#### Carbon dioxide transport

**Figure 2:**
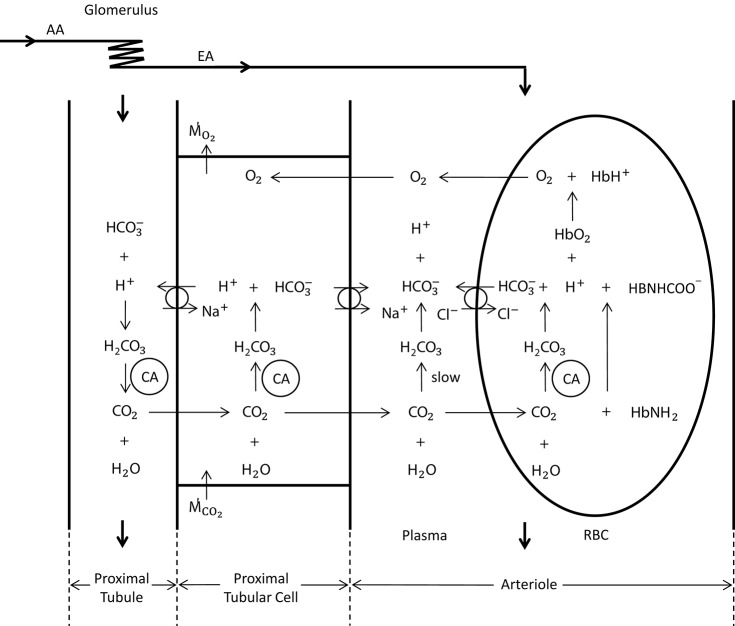
**Schematic representation of oxygen and carbon dioxide transport dynamics in the proximal tubule, proximal tubular cell and an arteriole (modified from Bidani et al., 1984; DuBose and Bidani, 1988; Huang and Hellums, 1994)**. The represented dynamics in the arteriole is also valid in the vessels of the preglomerular vasculature. In the vessels, CO_2_ is carried as CO_2_ in plasma and RBC (8%), ${\text{HCO}}_{3}^{-}$ in plasma and RBC (81%) and hemoglobin bound CO_2_ (11%) (Huang and Hellums, 1994). The CO_2_ hydration reaction in plasma is relatively slow, whereas in RBCs it is very fast due to the presence of carbonic anhydrase (CA). The anion transporter on the RBC membrane acts to exchange ${\text{HCO}}_{3}^{-}$ and Cl^−^ between plasma and RBC. To solve for carbon dioxide transport in vessels, seven species, i.e., CO_2_, ${\text{H}}_{\text{RBC}}^{+}$, ${\text{HCO}}_{3,\text{RBC}}^{-}$, ${\text{Cl}}_{\text{RBC}}^{-}$, ${\text{H}}_{\text{P}}^{+}$, ${\text{HCO}}_{3,\text{P}}^{-}$, and ${\text{Cl}}_{\text{P}}^{-}$, and their interactions need to be considered. In the proximal tubular cell, oxygen is consumed (${\dot{\text{M}}}_{{\text{O}}_{2}}$) and carbon dioxide is produced (${\dot{\text{M}}}_{{\text{CO}}_{2}}$). CO_2_ is rapidly turned into H^+^ and ${\text{HCO}}_{3}^{-}$ (due to carbonic anhydrase), for which the H^+^ is transported into the tubule to reabsorb the tubular ${\text{HCO}}_{3}^{-}$ in CO_2_ form. This cyclic mechanism functions to reabsorb the tubular bicarbonate as well as to generate bicarbonate that is transported back to the systemic blood (in opposition to proton excretion into the urine, which is buffered by phosphate, creatinine, urate, etc) to preserve acid—base homeostasis of the blood. The CO_2_ production in the proximal tubular cell is transported into the arterioles in CO_2_ and ${\text{HCO}}_{3}^{-}$ form, for which the ratio is unknown.

For the carbon dioxide transport, we base our calculations on a modified version of the model presented by Huang and Hellums (1994), accounting, next to oxygen, also for the following seven species: CO_2_, ${\text{H}}_{\text{RBC}}^{+}$, ${\text{HCO}}_{3,\text{RBC}}^{-}$, ${\text{Cl}}_{\text{RBC}}^{-}$, ${\text{H}}_{\text{P}}^{+}$, ${\text{HCO}}_{3,\text{P}}^{-}$ and ${\text{Cl}}_{\text{P}}^{-}$, namely carbon dioxide as well as hydrogen, bicarbonate and chloride ions in RBC and in plasma, respectively (see Figure 2). In RBCs, we assume chemical equilibrium for the carbon dioxide hydration/dehydration reaction, whereas in plasma we take the reaction term explicitly into account. We present below the seven governing equations for these species in the RBC-rich region and refer the reader to the Data Sheet 1 in Supplementary Material for their detailed derivation:

(7) $$\begin{array}{l} {}[\alpha_{{\text{CO}}_{2}}^{\text{P}}\left(1\;-\text{ h}\left(\text{r}\right)\right){\text{u}}_{\text{P}}\left(\text{r}\right)\\ +\;\alpha_{{\text{CO}}_{2}}^{\text{RBC}}\text{h}\left(\text{r}\right){\text{u}}_{\text{RBC}}\left(\text{r}\right)\;+\;\alpha_{{\text{CO}}_{2}}^{\text{RBC}}\text{h}\left(\text{r}\right){\text{u}}_{\text{RBC}}\left(\text{r}\right)\frac{\partial {\text{C}}_{{\text{HbCO}}_{2,\text{T}}}}{\partial {\text{C}}_{{\text{CO}}_{2}}^{\text{RBC}}}\\ +\;\alpha_{{\text{CO}}_{2}}^{\text{RBC}}\text{h}\left(\text{r}\right){\text{u}}_{\text{RBC}}\left(\text{r}\right){\text{K}}^{\prime ,\text{ RBC}}{\text{f}}_{\text{water}}\frac{\beta_{\text{RBC}}}{{\text{C}}_{{\text{H}}^{\;+\;}}^{\text{RBC}}(2.{303\text{C}}_{{\text{HCO}}_{3}^{-}}^{\text{RBC}}\;+\;\beta_{\text{RBC}})}]\\ \frac{\partial {\text{P}}_{{\text{CO}}_{2}}}{\partial \text{z}}\;=\;\frac{\;\alpha_{{\text{CO}}_{2}}^{\text{P}}{\text{D}}_{{\text{CO}}_{2}}^{\text{P}}}{\text{r}}\frac{\partial }{\partial \text{r}}\left(\text{r}\frac{\partial {\text{P}}_{{\text{CO}}_{2}}}{\partial \text{r}}\right)\;-\;\left(1\;-\text{ h}\left(\text{r}\right)\right){\text{R}}_{{\text{HCO}}_{3}^{\;-\;}}^{\text{P}}+\text{ h}\left(\text{r}\right){\text{J}}_{{\text{HCO}}_{3}^{-}}{\left[\frac{\text{s}}{\text{v}}\right]}_{\text{RBC}}, \end{array}$$

(8) $${\text{C}}_{{\text{HCO}}_{3}^{-}}^{\text{RBC}}=\beta_{\text{RBC}}\;\log\;{\text{C}}_{{\text{H}}^{+}}^{\text{RBC}}\;+\text{ b },$$

(9) $$(\beta_{\text{RBC}}\log\;{\text{C}}_{{\text{H}}^{+}}^{\text{RBC}}\;+\text{ b }){\text{C}}_{{\text{H}}^{+}}^{\text{RBC}}\;=\;{\text{K}}^{\prime ,\text{ RBC}}\;{\text{f}}_{\text{water}}\;{\text{C}}_{{\text{CO}}_{2}}^{\text{RBC}},$$

(10) $$\text{h}\left(\text{r}\right){\text{u}}_{\text{RBC}}\left(\text{r}\right)\frac{\partial {\text{C}}_{{\text{Cl}}^{-}}^{\text{RBC}}}{\partial \text{z}}\;=\;-\text{ h}\left(\text{r}\right){\text{J}}_{{\text{HCO}}_{3}^{-}}{\left[\frac{\text{s}}{\text{v}}\right]}_{\text{RBC}},$$

(11) $$\begin{array}{l} \left(1-\text{h}\left(\text{r}\right)\right){\text{u}}_{\text{P}}\left(\text{r}\right)\frac{\partial {\text{C}}_{{\text{Cl}}^{-}}^{\text{P}}}{\partial \text{z}}=\frac{{\text{D}}_{{\text{Cl}}^{-}}^{\text{P}}}{\text{r}}\frac{\partial }{\partial \text{r}}\left(\text{r}\frac{\partial {\text{C}}_{{\text{Cl}}^{-}}^{\text{P}}}{\partial \text{r}}\right)\\ \text{ + h}\left(\text{r}\right){\text{J}}_{{\text{HCO}}_{3}^{-}}{\left[\frac{\text{s}}{\text{v}}\right]}_{\text{RBC}}, \end{array}$$

(12) $$\begin{array}{l} \left(1-\text{h}\left(\text{r}\right)\right){\text{u}}_{\text{P}}\left(\text{r}\right)\frac{\partial {\text{C}}_{{\text{H}}^{+}}^{\text{P}}}{\partial \text{z}}=\frac{{\text{D}}_{{\text{H}}^{+}}^{\text{P}}}{\text{r}}\frac{\partial }{\partial \text{r}}\left(\text{r}\frac{\partial {\text{C}}_{{\text{H}}^{+}}^{\text{P}}}{\partial \text{r}}\right)\\ \;+\;\frac{2.{303\text{C}}_{{\text{H}}^{+}}^{\text{P}}}{\beta_{\text{P}}}\left(1-\text{h}\left(\text{r}\right)\right){\text{R}}_{{\text{HCO}}_{3}^{-}}^{\text{P}}, \end{array}$$

(13) $$\begin{array}{l} \left(1-\text{h}\left(\text{r}\right)\right){\text{u}}_{\text{P}}\left(\text{r}\right)\frac{\partial {\text{C}}_{{\text{HCO}}_{3}^{-}}^{\text{P}}}{\partial \text{z}}=\frac{{\text{D}}_{{\text{HCO}}_{3}^{-}}^{\text{P}}}{\text{r}}\frac{\partial }{\partial \text{r}}\left(\text{r}\frac{\partial {\text{C}}_{{\text{HCO}}_{3}^{-}}^{\text{P}}}{\partial \text{r}}\right)\\ \;+\left(1-\text{h}\left(\text{r}\right)\right){\text{R}}_{{\text{HCO}}_{3}^{-}}^{\text{P}}-\text{ h}\left(\text{r}\right){\text{J}}_{{\text{HCO}}_{3}^{-}}{\left[\frac{\text{s}}{\text{v}}\right]}_{\text{RBC}},\; \end{array}$$

where $\alpha_{{\text{CO}}_{2}}^{\text{P}}$ and $\alpha_{{\text{CO}}_{2}}^{\text{RBC}}$ are the solubility of carbon dioxide in plasma and inside the RBC, respectively. K′^RBC^ and f_water_ are the apparent dissociation constant for H_2_CO_3_ inside RBC and water fraction of RBC volume, respectively. β_P_ and β_RBC_ are the buffering capacity in plasma and in RBC, respectively. Finally, ${\left[\frac{\text{s}}{\text{v}}\right]}_{\text{RBC}}$ is the surface to volume ratio of an RBC. Equation (8) represents the normal non-${\text{HCO}}_{3}^{-}$ titration line in the Davenport diagram with buffering capacity β_RBC_ as slope and b as a constant, which is calculated based on systemic arterial blood values of ${\text{C}}_{{\text{HCO}}_{3}^{-}}^{\text{RBC}}=6.52\text{mM}$ and pH_RBC_ = 7.24 (Davenport, 1958; Boron and Boulpaep, 2012). The permeability of carbon dioxide in plasma is defined as ${\text{K}}_{{\text{CO}}_{2}}^{\text{P}}=\alpha_{{\text{CO}}_{2}}^{\text{P}}{\text{D}}_{{\text{CO}}_{2}}^{\text{P}}$, where ${\text{D}}_{{\text{CO}}_{2}}^{\text{P}}$ is the diffusion coefficient of carbon dioxide in plasma, whereas ${\text{D}}_{{\text{Cl}}^{-}}^{\text{P}}$, ${\text{D}}_{{\text{H}}^{+}}^{\text{P}}$, and ${\text{D}}_{{\text{HCO}}_{3}^{-}}^{\text{P}}$ are the diffusion coefficients of chloride, hydrogen and bicarbonate ions, respectively. ${\text{R}}_{{\text{HCO}}_{3}^{-}}^{\text{P}}$ and ${\text{J}}_{{\text{HCO}}_{3}^{-}}$ are the bicarbonate formation rate in plasma and the anion transporter flux through the RBC membrane (see Data Sheet 1 in Supplementary Material for their calculation). In the RBC-free region, plasma carbon dioxide, hydrogen ion, bicarbonate ion and chloride ion concentration follow, respectively:

(14) $$\left[\alpha_{{\text{CO}}_{2}}^{\text{P}}{\text{u}}_{\text{p}}^{\prime }(\text{r})\right]\frac{\partial {\text{P}}_{{\text{CO}}_{2}}}{\partial \text{z}}=\frac{\alpha_{{\text{CO}}_{2}}{\text{D}}_{{\text{CO}}_{2}}^{\text{P}}}{\text{r}}\frac{\partial }{\partial \text{r}}\left(\text{r}\frac{\partial {\text{P}}_{{\text{CO}}_{2}}}{\partial \text{r}}\right)\;-\;{\text{R}}_{{\text{HCO}}_{3}^{-}}^{\text{P}},$$

(15) $${\text{u}}_{\text{p}}^{\prime }(\text{r})\frac{\partial {\text{C}}_{{\text{H}}^{+}}^{\text{P}}}{\partial \text{z}}=\frac{{\text{D}}_{{\text{H}}^{+}}^{\text{P}}}{\text{r}}\frac{\partial }{\partial \text{r}}\left(\text{r}\frac{\partial {\text{C}}_{{\text{H}}^{+}}^{\text{P}}}{\partial \text{r}}\right)\;+\;\frac{2.{303\text{C}}_{{\text{H}}^{+}}^{\text{P}}}{\beta_{\text{P}}}{\text{R}}_{{\text{HCO}}_{3}^{-}}^{\text{P}},$$

(16) $${\text{u}}_{\text{p}}^{\prime }(\text{r})\frac{\partial {\text{C}}_{{\text{HCO}}_{3}^{-}}^{\text{P}}}{\partial \text{z}}=\frac{{\text{D}}_{{\text{HCO}}_{3}^{-}}^{\text{P}}}{\text{r}}\frac{\partial }{\partial \text{r}}\left(\text{r}\frac{\partial {\text{C}}_{{\text{HCO}}_{3}^{-}}^{\text{P}}}{\partial \text{r}}\right)\;+\;{\text{R}}_{{\text{HCO}}_{3}^{-}}^{\text{P}},$$

(17) $${\text{u}}_{\text{p}}^{\prime }\left(\text{r}\right)\frac{\partial {\text{C}}_{{\text{Cl}}^{-}}^{\text{P}}}{\partial \text{z}}=\frac{{\text{D}}_{{\text{Cl}}^{-}}^{\text{P}}}{\text{r}}\frac{\partial }{\partial \text{r}}\left(\text{r}\frac{\partial {\text{C}}_{{\text{Cl}}^{-}}^{\text{P}}}{\partial \text{r}}\right).$$

The vessel walls are assumed impermeable to hydrogen, bicarbonate and chloride ions, since these require active transport, which is not considered significant in the preglomerular vasculature. Consequently, in the tissue, only carbon dioxide diffusion is considered. The tissue carbon dioxide partial pressure, P_CO_2__, is governed by:

(18) $$\alpha_{{\text{CO}}_{2}}^{\text{T}}{\text{D}}_{{\text{CO}}_{2}}^{\text{T}}\nabla^{2}{\text{P}}_{{\text{CO}}_{2}}\;=\;-\;\left(1-\;\varphi \right)({\dot{\text{M}}}_{{\text{CO}}_{2}}\;+\;{\dot{\text{M}}}_{\text{c},\;{\text{CO}}_{2}}),$$

where $\alpha_{{\text{CO}}_{2}}^{\text{T}}$ is the solubility of carbon dioxide in tissue. The permeability of carbon dioxide in tissue is defined as ${\text{K}}_{{\text{CO}}_{2}}^{\text{T}}=\alpha_{{\text{CO}}_{2}}^{\text{T}}{\text{D}}_{{\text{CO}}_{2}}^{\text{T}}$, where ${\text{D}}_{{\text{CO}}_{2}}^{\text{T}}$ is the diffusion coefficient of carbon dioxide in tissue. ${\dot{\text{M}}}_{{\text{CO}}_{2}}\text{and }{\dot{\text{M}}}_{\text{c},\;{\text{CO}}_{2}}$ are the metabolic carbon dioxide production rate in the tissue and capillary carbon dioxide source/sink term, respectively. Here we assume that all CO_2_ produced in the tissue is taken up by the capillaries, i.e., ${\dot{\text{M}}}_{\text{c},\;{\text{CO}}_{2}}$ = ${-\dot{\text{M}}}_{{\text{CO}}_{2}}$, as explained in the next section. All parameters related to oxygen and carbon dioxide transport dynamics are given in Table 2.

**Table 2:** **Parameters used in the study**.

**Parameter**	**Value**	**References**
C_HbT_	19.01 mol/m^3^	Present study
${\text{D}}_{{\text{Cl}}^{\text{-}}}^{\text{P}}$	1.29 · 10^−9^ m^2^/s	Huang and Hellums, 1994
${\text{D}}_{{\text{CO}}_{2}}^{\text{P}}$	1.76 · 10^−9^ m^2^/s	Huang and Hellums, 1994
${\text{D}}_{{\text{CO}}_{2}}^{\text{T}}$	1.76 · 10^−9^ m^2^/s	Huang and Hellums, 1994
${\text{D}}_{{\text{H}}^{\text{+}}}^{\text{P}}$	9.52 · 10^−9^ m^2^/s	Huang and Hellums, 1994
${\text{D}}_{{\text{HCO}}_{3}^{\text{-}}}^{\text{P}}$_3_	1.25 · 10^−9^ m^2^/s	Huang and Hellums, 1994
${\text{D}}_{{\text{O}}_{2}}^{\text{P}}$	2.75 · 10^−9^ m^2^/s	Nair et al., 1989, 1990
${\text{D}}_{{\text{O}}_{2}}^{\text{T}}$	2.41 · 10^−9^ m^2^/s	Vadapalli et al., 2002; Moschandreou et al., 2011
f_water_	0.72	Huang and Hellums, 1994
K_DPG_	1.0 mM^−1^	Huang and Hellums, 1994
K′, ^RBC^	5.01 · 10^−7^ M	Huang, 1991
${\text{k}}_{\text{u}}^{\text{P}}$	0.134 s	Huang, 1991
${\text{k}}_{\text{v}}^{\text{P}}$	57.5 s	Huang, 1991)
${\text{K}}_{1}^{\text{P}}$	3.5 · 10^−4^ M	Huang, 1991
k_trans_	5.0 · 10^4^ s	Huang and Hellums, 1994
K_A_	0.2 mM^−1^	Huang and Hellums, 1994
R_RBC_	4 μm	Nair et al., 1989
t_RBC_	1.3 μm	Nair et al., 1989
T_tot_	1.0 · 10^6^	Huang and Hellums, 1994
s_RBC_	163 · 10^−12^ m^2^	Huang and Hellums, 1994
$\left[\frac{\text{s}}{\text{v}}\right]\text{RBC}$	1.87 μm^−1^	Huang and Hellums, 1994
u	1.85 mm/s	Jeong et al., 2006
$\alpha_{{\text{O}}_{2}}^{\text{P}}$	1.10 μM/mmHg	Christoforides et al., 1969; Vadapalli et al., 2002
$\alpha_{{\text{O}}_{2}}^{\text{RBC}}$	1.33 μM/mmHg	Nair et al., 1990; Vadapalli et al., 2002
$\alpha_{{\text{O}}_{2}}^{\text{T}}$	1.53 μM/mmHg	Vadapalli et al., 2002; Moschandreou et al., 2011
$\alpha_{{\text{CO}}_{2}}^{\text{P}}$	30.3 μM/mmHg	Huang and Hellums, 1994
$\alpha_{{\text{CO}}_{2}}^{\text{RBC}}$	26 μM/mmHg	Huang and Hellums, 1994
$\alpha_{{\text{CO}}_{2}}^{\text{T}}$	30.3 μM/mmHg	Huang and Hellums, 1994
β_RBC_	60 mM H^+^/pH	Huang and Hellums, 1994
β_P_	5.77 mM H^+^/pH	Huang and Hellums, 1994
λ_α_	0.65 mM^−1^	Huang and Hellums, 1994
λ_β_	0.24 mM^−1^	Huang and Hellums, 1994

*C_HbT_, total heme group concentration; $D_{{Cl}^{-}}^{P}$, $D_{{CO}_{\text{2}}}^{P}$, $D_{H^{+}}^{P}$, $D_{{HCO}_{\text{3}}^{-}}^{P}$ and $D_{O_{\text{2}}}^{P}$, diffusion coefficients of chloride ion, carbon dioxide, hydrogen ion, bicarbonate ion, and oxygen in plasma, respectively; $D_{{CO}_{\text{2}}}^{T}$ and $D_{O_{\text{2}}}^{T}$, diffusion coefficients of carbon dioxide, and oxygen in tissue, respectively; f_water_, water fraction of total RBC volume; K_DPG_, association constant for DPG and hemoglobin; K′^, RBC^, apparent dissociation constant for H_2_CO_3_ in RBC; $k_{u}^{P}$, $k_{v}^{P}$ and $K_{1}^{P}$, CO_2_ hydration reaction rate, H_2_CO_3_ dehydration rate constant and first dissociation constant for H_2_CO_3_ in plasma, respectively; k_trans_ and K_A_, translocation and equilibrium association rate constants, respectively; R_RBC_, radius of RBC disk; t_RBC_, maximum half thickness of RBC; T_tot_, total number of anion transporters on RBC membrane; s_RBC_ and v_RBC_, surface area and volume of RBC; u, oxygen advection velocity in capillaries; $\alpha_{O_{\text{2}}}^{P}$, $\alpha_{O_{\text{2}}}^{RBC}$ and $\alpha_{O_{\text{2}}}^{T}$, solubility of oxygen in plasma, RBC and tissue, respectively; $\alpha_{{CO}_{\text{2}}}^{P}$, $\alpha_{CO_{\text{2}}}^{RBC}$, and $\alpha_{CO_{\text{2}}}^{T}$, solubility of carbon dioxide in plasma, RBC and tissue, respectively; β_RBC_ and β_P_, buffering capacity in RBC and in plasma, respectively; λ_α_ and λ_β_, association constant for CO_2_ binding to the α- and β-chains of hemoglobin*.

### Computational implementation

Initially, the advection path of oxygen along capillaries, the hematocrit profile and the plasma and RBC velocity profiles are determined as described in Olgac and Kurtcuoglu (2015a) and set on the 3D computational domain. With all flow fields set, Equations (7)–(17) are solved inside the vessels while Equation (18) is solved in the tissue for carbon dioxide transport. The solutions of these two sets of equations are determined concurrently in the 3D computational domain. Once the solutions have been obtained, Equations (1)–(3) are solved in the same 3D domain for oxygen transport either with a constant P_50_ or with a variable P_50_ that is dependent on the local P_CO_2__ and pH. The computations are performed in a time-dependent manner using the Euler method for time discretization. The computations are terminated when steady-state is reached, i.e., when temporal changes in the P_CO_2__ and pH profiles (for carbon dioxide transport calculations) and in the P_O_2__ profile (for oxygen transport calculations) at each representative level become negligible. A first order upwind scheme and a second order Gaussian integration scheme with harmonic interpolation for oxygen and carbon dioxide permeability are used for the spatial discretization of divergence and Laplacian terms, respectively. The resulting algebraic system is solved using a pre-conditioned bi-conjugate gradient solver (Ferzinger and Periæ, 1998) in Foam-extend-3.1 (Weller et al., 1998; Jasak et al., 2007). The solution is determined on all representative levels simultaneously. Independence of the reported results from the employed spatial discretization was confirmed as detailed in the Data Sheet 1 in Supplementary Material.

#### Boundary conditions

P_O_2__ at the inlet of the renal artery is fixed to P_O_2_, RA_. For carbon dioxide transport, at the renal artery inlet, chemical equilibrium of the carbon dioxide hydration/dehydration reaction both in RBC as well as in plasma (${\text{R}}_{{\text{HCO}}_{3}^{-},\text{inlet},\text{a},10}^{\text{P}}=0$) and zero flux of bicarbonate and chloride ions over the RBC membrane (${\text{J}}_{{\text{HCO}}_{3}^{-},\text{inlet},\text{ a},10}=0$) are assumed, since the renal artery receives fresh systemic blood from the aorta. The inlet boundary condition (BC) values based on systemic arterial blood are listed in Table 3.

**Table 3:** **Renal artery inlet boundary condition values**.

**Parameter**	**Value**	**References**
P_CO_2__	40 mmHg	Dash et al., 2016
pH_RBC_	7.24	Dash et al., 2016
${\text{C}}_{{\text{HCO}}_{3}^{\text{-}}}^{\text{RBC}}$	6.52 mM	Present study
${\text{C}}_{{\text{Cl}}^{\text{-}}}^{\text{RBC}}$	26.37 mM	Present study
pH_P_	7.4	Dash et al., 2016
${\text{C}}_{{\text{HCO}}_{3}^{\text{-}}}^{\text{P}}$	24.72 mM	Present study
${\text{C}}_{{\text{Cl}}^{\text{-}}}^{\text{P}}$	100 mM	Present study
P_O_2__	79 mmHg	Welch et al., 2001

*P_CO_2__, carbon dioxide partial pressure; P_O_2__, oxygen partial pressure; pH_RBC_, pH in RBC; pH_P_, pH in plasma; $C_{{HCO}_{\text{3}}^{-}}^{RBC}$, bicarbonate ion concentration in RBC; $C_{{Cl}^{-}}^{RBC}$, chloride ion concentration in RBC; $C_{{HCO}_{\text{3}}^{-}}^{P}$, bicarbonate ion concentration in plasma; $C_{{Cl}^{-}}^{P}$, chloride ion concentration in plasma. $C_{{HCO}_{\text{3}}^{-}}^{P}$ and $C_{{HCO}_{\text{3}}^{-}}^{RBC}$ are calculated from chemical equilibrium with P_CO_2__ = 40 mmHg, pH_RBC_ = 7.24 and pH_P_ = 7.4. $C_{{Cl}^{-}}^{P}$ is set to a standard value and $C_{{Cl}^{-}}^{RBC}$ is specified such that $J_{{HCO}_{\text{3}}^{-}}=0$, i.e., $\frac{C_{{HCO}_{\text{3}}^{-}}^{RBC}}{C_{{HCO}_{\text{3}}^{-}}^{P}}=\frac{C_{{Cl}^{-}}^{RBC}}{C_{{Cl}^{-}}^{P}}$. The exact values of $C_{{Cl}^{-}}^{RBC}$ and $C_{{Cl}^{-}}^{P}$ are not important, but their ratio is, as it determines the driving force for transmembrane transport. Chloride ions are not involved in any other reaction but the exchange of chloride with bicarbonate ions over the RBC membrane (Huang, 1991)*.

For the rest of the inlet BCs, at each representative level throughout the computations, partial pressure of oxygen at the inlet, P_O_2_, inlet, i_, is specified such that the oxygen delivery D_O_2_, inlet, i_ (see Data Sheet 1 in Supplementary Material for calculation) matches the delivery at the outlet of the previous level:

(19) $$\begin{array}{l} {\text{D}}_{{\text{O}}_{2},\text{inlet},\text{a},\text{i}}={\text{D}}_{{\text{O}}_{2},\text{outlet},\text{a},\text{i}+1}\text{ for arteries},\\ \;{\text{D}}_{{\text{O}}_{2},\text{inlet},\text{v},\text{i}}={\text{D}}_{{\text{O}}_{2},\text{outlet},\text{v},\text{i}-1}\text{ for veins}. \end{array}$$

For carbon dioxide transport, at each representative level throughout the computational domain, partial pressure of carbon dioxide at the inlet, P_CO_2_, inlet, i_, hydrogen ion concentration in RBC at the inlet, ${\text{C}}_{{\text{H}}^{+},\text{inlet},\text{i}}^{\text{RBC}}$, and bicarbonate ion concentration in RBC at the inlet, ${\text{C}}_{{\text{HCO}}_{3}^{-},\text{inlet},\text{i}}^{\text{RBC}}$, are specified such that a new chemical equilibrium is established in RBC at the inlet, with carbon dioxide delivery D_CO_2_, inlet, i_ matching the delivery at the outlet of the previous level:

(20) $$\begin{array}{l} {\text{D}}_{{\text{CO}}_{2},\text{inlet},\text{a},\text{i}}={\text{D}}_{{\text{CO}}_{2},\text{outlet},\text{a},\text{i}+1}\text{ for arteries},\\ {\text{D}}_{{\text{CO}}_{2},\text{inlet},\text{v},\text{i}}={\text{D}}_{{\text{CO}}_{2},\text{outlet},\text{v},\text{i}-1}\text{ for veins}, \end{array}$$

where D_CO_2_, inlet, i_ is the sum of deliveries of CO_2_ in plasma and RBC, hemoglobin-bound CO_2_ and ${\text{HCO}}_{3}^{-}$ in RBC (see Data Sheet 1 in Supplementary Material for calculation).

The remaining boundary conditions, i.e., concentration of chloride ion in RBC at the inlet, ${\text{C}}_{{\text{Cl}}^{-},\text{inlet},\text{i}}^{\text{RBC}}$, concentration of hydrogen ion in plasma at the inlet, ${\text{C}}_{{\text{H}}^{+},\text{inlet},\text{i}}^{\text{P}}$, concentration of bicarbonate ion in plasma at the inlet, ${\text{C}}_{{\text{HCO}}_{3}^{-},\text{inlet},\text{i}}^{\text{P}}$, and concentration of chloride ion in plasma at the inlet, ${\text{C}}_{{\text{Cl}}^{-},\text{inlet},\text{i}}^{\text{P}}$, are specified such that the delivery D_inlet, i_ of the respective species (see Data Sheet 1 in Supplementary Material for calculation) matches the delivery at the outlet of the previous level:

(21) $$\begin{array}{l} {\text{D}}_{\text{inlet},\text{a},\text{i}}={\text{D}}_{\text{outlet},\text{a},\text{i}+1}\text{ for arteries},\\ {\text{D}}_{\text{inlet},\text{v},\text{i}}={\text{D}}_{\text{outlet},\text{v},\text{i}-1}\text{ for veins}. \end{array}$$

The venous return P_O_2_, inlet, v, 0_ is specified such that:

(22) $${\text{D}}_{{\text{O}}_{2},\text{inlet},\text{v},0}={\text{D}}_{{\text{O}}_{2},\text{outlet},\text{a},0}+{\dot{\text{V}}}_{{\text{O}}_{2},\text{M}}-{\text{J}}_{{\text{O}}_{2},\text{c}},$$

where ${\dot{\text{V}}}_{{\text{O}}_{2},\text{M}}$ and J_O_2_, c_ are the medullary oxygen consumption rate and the flux of oxygen between the capillaries and the cortical tissue, respectively.

To set the boundary conditions on the venous return for carbon dioxide transport, we consider the CO_2_ transport dynamics in the vicinity of a proximal tubular cell as shown in Figure 2. Here we assume that all cortical CO_2_ production is due to active transport by the tubules, as the contribution of basal metabolism is small [3–18% in the mammalian kidney (Cohen and Kamm, 1981)]. We therefore further assume that all of the CO_2_ produced in the cortex is taken up by the nearby capillaries and transported to the venous return (thus does not diffuse into arterioles in the vicinity), where CO_2_ produced in the medulla is added as well. The bicarbonate generation rate required to preserve acid-base homeostasis of the blood is approximately 1 mmol/kg body weight (Boron and Boulpaep, 2012), or 0.19 μmol/min for a Wistar Kyoto (WKY) rat. We neglect this bicarbonate generation, as its rate is much smaller than the overall carbon dioxide production rate (see Table 4). CO_2_ produced by the proximal tubular cell is transported into the nearby capillaries in both CO_2_ and bicarbonate form, for which the ratio is unknown. Their ratio on the venous return is also unknown since they are subject to further reactions on the path from the capillaries to the venous return. We therefore consider two extreme conditions on the venous return and assume that the real state lies somewhere between these two conditions (see Data Sheet 1 in Supplementary Material for details):

*Condition 1*: Balanced CO_2_ distribution on the venous return. This condition assumes that the total renal CO_2_ production is distributed into all forms of CO_2_ on venous return, i.e., CO_2_ in plasma and RBC, ${\text{HCO}}_{3}^{-}$ in plasma and RBC, and hemoglobin bound CO_2_.

*Condition 2*: Unbalanced CO_2_ distribution on the venous return. This condition assumes that the total renal CO_2_ production is distributed into all forms of CO_2_ except ${\text{HCO}}_{3}^{-}$ in plasma on venous return.

**Table 4:** **Base case values**.

**Value**	**Base case**	**References**
P_O_2_, RA_	79 mmHg	Welch et al., 2001
P_CO_2_, RA_	40 mmHg	Dash et al., 2016
pH_P, RA_	7.4	Dash et al., 2016
pH_RBC, RA_	7.24	Dash et al., 2016
RBF	5.61 mL/min	Welch et al., 2001
$\dot{\text{D}}{\text{O}}_{2},RA$	46.09 μmol/min	Welch et al., 2001
$\dot{\text{D}}\text{C}{\text{O}}_{2},T,RA$	109.79 μmol/min	Present study
$\dot{\text{V}}{\text{O}}_{2}$	−8.36 μmol/min	Welch et al., 2001
$\dot{\text{V}}\text{C}{\text{O}}_{2}$	6.69 μmol/min	Present study

*P_O_2_, RA_, P_CO_2_, RA_, pH_P, RA_ and pH_RBC, RA_, partial pressure of oxygen and carbon dioxide and pH in plasma and RBC in the renal artery, respectively; RBF, renal blood flow; Ḋ_O_2_, RA_, renal artery oxygen delivery; Ḋ_CO_2_, T, RA_, renal artery total carbon dioxide delivery; ${\dot{V}}_{O_{\text{2}}}$, oxygen consumption rate; ${\dot{V}}_{CO_{2}}$, carbon dioxide production rate*.

**Figure 3:**
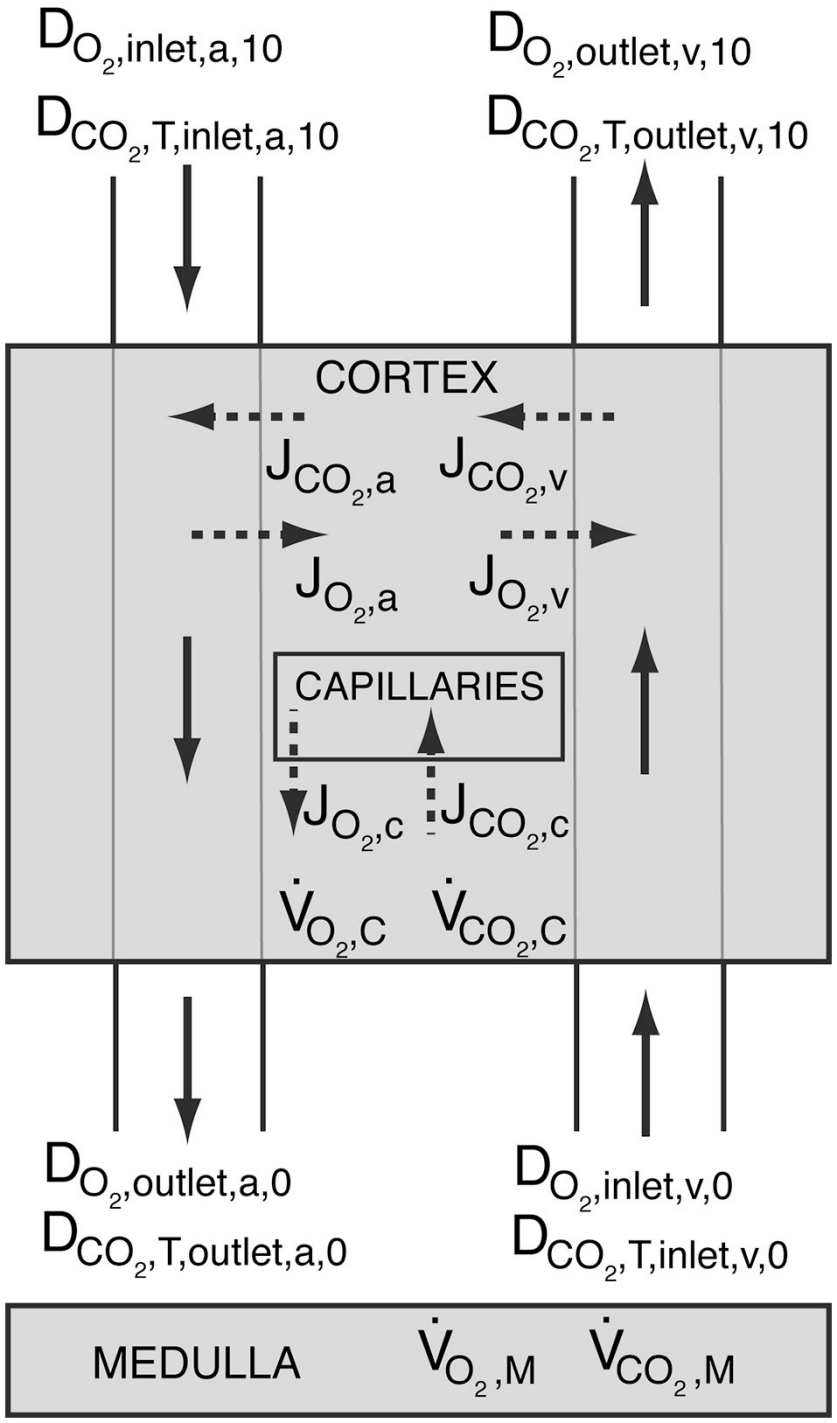
**Schematic showing oxygen delivery (D_**O**_**2**__) and total carbon dioxide delivery (D_**CO**_**2**_, **T**_) throughout the kidney**. The shaded regions represent the cortex and the medulla. Solid arrows symbolize blood flow, whereas dashed arrows represent oxygen and carbon dioxide flux. Blood enters the arterial tree at order 10 and exits the explicitly modeled cortical domain at order 0. Blood enters cortical domain again at the venous side at order 0 and exits at order 10. J_O_2_, a_, J_O_2_, v_ and J_O_2_, c_, and J_CO_2_, a_, J_CO_2_, v_ and J_CO_2_, c_ are the oxygen and carbon dioxide fluxes between the arteries and the tissue, the veins and the tissue, and capillaries and the tissue, respectively. Positive values designate flux from vessels to the tissue, whereas negative values stand for oxygen or carbon dioxide transfer from the tissue into the vessels. ${\dot{\text{V}}}_{{\text{O}}_{2},\text{C}}$ and ${\dot{\text{V}}}_{{\text{O}}_{2},\text{M}}$ are the cortical and medullary oxygen consumption rates, respectively, whereas ${\dot{\text{V}}}_{\text{C}{\text{O}}_{2},\text{C}}$ and ${\dot{\text{V}}}_{{\text{CO}}_{2},\text{M}}$ are the cortical and medullary carbon dioxide production rates, respectively. All carbon dioxide produced in the cortex is assumed to be taken up by capillaries, i.e., ${\text{J}}_{\text{C}{\text{O}}_{2},\text{c}}={\dot{\text{V}}}_{\text{C}{\text{O}}_{2},\text{C}}$.

The above given BCs ensure for both conditions that total oxygen and carbon dioxide delivery throughout the kidney is conserved, i.e., ${\text{D}}_{{\text{O}}_{2},\text{inlet},\text{a},10}={\text{D}}_{{\text{O}}_{2},\text{outlet},\text{v},10}-{\dot{\text{V}}}_{{\text{O}}_{2},\text{C}}-{\dot{\text{V}}}_{{\text{O}}_{2},\text{M}}$ and ${\text{D}}_{{\text{CO}}_{2},\text{T},\text{inlet},\text{a},10}={\text{D}}_{\text{C}{\text{O}}_{2},\text{T},\text{outlet},\text{v},10}-{\dot{\text{V}}}_{\text{C}{\text{O}}_{2},\text{C}}-{\dot{\text{V}}}_{\text{C}{\text{O}}_{2},\text{M}}$ (see Figure 3). D_CO_2_, T_ is the total carbon dioxide delivery, which is the sum of deliveries of CO_2_ in plasma and RBC, hemoglobin-bound CO_2_ and ${\text{HCO}}_{3}^{-}$ in plasma and RBC (see Data Sheet 1 in Supplementary Material for calculation).

As to the outlet BCs, convective flux boundary conditions are imposed on all vessel outlets by setting the diffusive flux to zero. On all lateral surfaces, diffusive flux is set to zero. At the vessel-tissue interfaces, there is continuity of oxygen and carbon dioxide partial pressure and oxygen and carbon dioxide flux such that:

(23a) $$\alpha_{{\text{O}}_{2}}^{\text{P}}{\text{D}}_{{\text{O}}_{2}}^{\text{P}}\frac{\partial {\text{P}}_{{\text{O}}_{2}}}{\partial \text{r}}=\alpha_{{\text{O}}_{2}}^{\text{T}}{\text{D}}_{{\text{O}}_{2}}^{\text{T}}\frac{\partial {\text{P}}_{{\text{O}}_{2}}}{\partial \text{r}},\text{ r}=\text{R},$$

(23b) $$\alpha_{{\text{CO}}_{2}}^{\text{P}}{\text{D}}_{{\text{CO}}_{2}}^{\text{P}}\frac{\partial {\text{P}}_{{\text{CO}}_{2}}}{\partial \text{r}}=\alpha_{{\text{CO}}_{2}}^{\text{T}}{\text{D}}_{{\text{CO}}_{2}}^{\text{T}}\frac{\partial {\text{P}}_{{\text{CO}}_{2}}}{\partial \text{r}},\text{ r}=\text{R}.$$

### Base case

The base case is derived from *in vivo* P_O_2__ measurements with oxygen-sensitive ultramicroelectrodes in normotensive WKY rats as reported by Welch et al. (2001). Relevant values for the base case are summarized in Table 4. Of these, renal blood flow (RBF), oxygen delivery (D_O_2__), oxygen consumption rate (${\dot{\text{V}}}_{{\text{O}}_{2}}$) and renal artery P_O_2__ (P_O_2_, RA_) are measured values (Welch et al., 2001), renal artery P_CO_2__ (P_CO_2_, RA_), pH_RBC, RA_ and pH_P, RA_ are based on systemic arterial blood (Dash et al., 2016), and carbon dioxide delivery (D_CO_2__) is calculated based on these values and the rest of the renal artery inlet boundary conditions presented in Table 3. To set the carbon dioxide production rate in dependence of the oxygen consumption rate, we assume a respiratory quotient (RQ) of 0.8 (Weidemann and Krebs, 1969; Burke et al., 1999; Dash and Bassingthwaighte, 2006). Prescribing the reference renal artery P_O_2__, P_O_2_, RA_, on the renal artery inlet, the total heme group concentration is set such that the renal artery oxygen delivery (D_O_2_, inlet, a, 10_) matches the values given in Table 4. Hence, the total heme group concentration is set to C_HbT_ = 19.01 mol/m^3^ for the base case. We set capillary P_O_2__, P_O_2_, c_, to an average of afferent arteriole outlet and venous return inlet, i.e., P_O_2_, c_ = 0.5(P_O_2_, outlet, a, 0_ + P_O_2_, inlet, v, 0_). See Olgac and Kurtcuoglu (2015a) for details on capillary P_O_2__.

## Results

### Base case under Conditions 1 and 2

**Figure 4:**
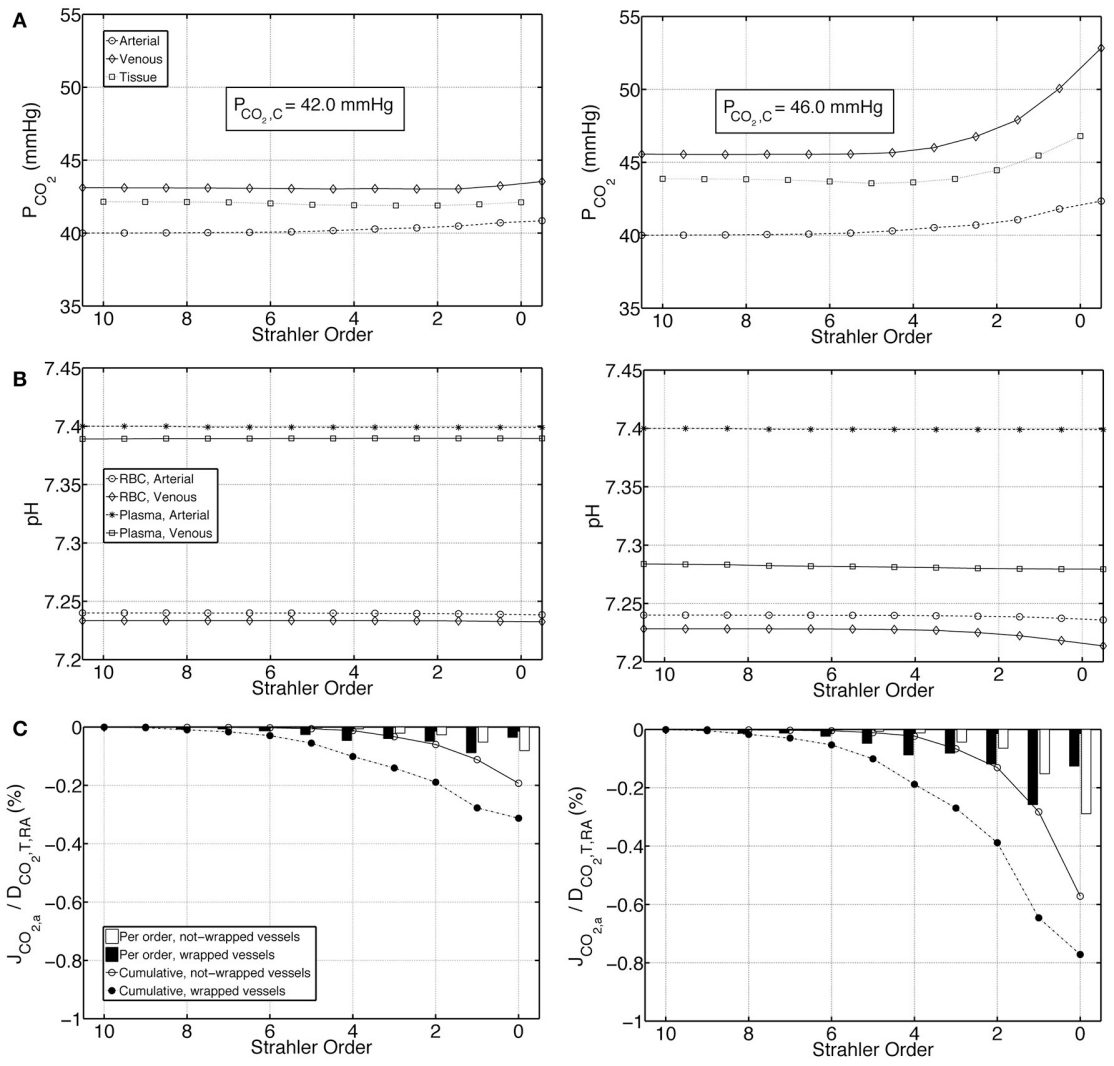
**Comparison of results for the base case under ***Condition 1*** (left) and ***Condition 2*** (right). (A)** Arterial, venous and tissue partial pressure of carbon dioxide (P_CO_2__) profiles, as well as the average cortical carbon dioxide partial pressure, P_CO_2_, C_, **(B)** Arterial and venous plasma and RBC pH profiles, **(C)** Carbon dioxide flux across artery walls, J_CO_2, a__, reported as percentage of total renal carbon dioxide delivery, D_CO_2_, T, RA_. Cumulative values as well as values for each individual order are shown. Positive flux represents flux from the vessel into the tissue, whereas negative flux denotes the opposite, i.e., negative J_CO_2_, a_ denotes carbon dioxide shunted to the arterial tree.

We first present the output of the model employing two different boundary conditions on the venous return. *Condition 1* distributes total renal CO_2_ production into all forms of CO_2_ in the venous return, whereas *Condition 2* excludes plasma bicarbonate in this distribution. Figures 4A,B demonstrate on the left panel under *Condition 1* and on the right panel under *Condition 2*, arterial, venous and tissue P_CO_2__ as well as plasma and RBC pH profiles, respectively. *Condition 1* results in flatter profiles of both P_CO_2__ and pH compared to *Condition 2*. Under *Condition 1*, P_CO_2__ increases from 40 mmHg at the inlet of the renal artery (order 10) to 40.8 mmHg at the outlet of the afferent arteriole (order 0). Under *Condition 2*, the increase is to 42.3 mmHg. On the venous return, under *Condition 1*, P_CO_2__ = 43.5 mmHg, whereas under *Condition 2*, P_CO_2__ = 52.8 mmHg. The lower increase in P_CO_2__ under *Condition 1* is due to the fact that CO_2_ production is distributed into all forms of CO_2_ on the venous return, including the dominant plasma bicarbonate. Conversely, as there is no plasma bicarbonate added to the venous return under *Condition 2*, the same rate of renal CO_2_ production results in a higher increase in P_CO_2__. This is also evident in the pH profiles: Under *Condition 1*, the pH profiles are almost flat with a slight overall decrease in plasma and RBC pH on the venous side due to the increased acidity (added renal CO_2_ production). Under *Condition 2*, plasma and RBC pH on the venous return are 7.28 and 7.21, respectively. This increased acidity is again owed to the addition of produced CO_2_ to the venous return in forms other than plasma bicarbonate. Figure 4C shows for both conditions the flux of carbon dioxide between artery walls and the tissue. As indicated by the negative fluxes, there is venous-to-arterial carbon dioxide shunting under both conditions, with more shunting under *Condition 2*. Overall, approximately 0.5 and 1.4% of the total renal carbon dioxide delivery is shunted under *Conditions 1 & 2*, respectively. The larger amount of carbon dioxide shunting under *Condition 2* is primarily due to the higher P_CO_2__ gradient between the venous and the arterial sides compared to *Condition 1*.

The investigated *Conditions 1* and *2*, i.e., the distribution of total renal CO_2_ in all forms of CO_2_ on the venous return and the exclusion of plasma bicarbonate during this distribution, respectively, represent extreme cases. The real state must lie between these two. We therefore estimate the average cortical P_CO_2__, P_CO_2_, C_, to be between 42.0 and 46.0 mmHg.

### Effects of CO_2_ transport on O_2_ shunting

We performed O_2_ transport calculations with a constant P_50_, and a variable P_50_ whose dependence on the local P_CO_2__ and pH is given by Equation 6(a)–(c). The O_2_ transport calculations with variable P_50_ are based on the P_CO_2__ and pH fields obtained from carbon dioxide transport calculations under *Condition 2*, because under this condition, the maximum possible increase in P_CO_2__ and decrease in pH in the preglomerular vasculature compared to the systemic arterial blood are reached. In other words, this condition captures the highest possible effect of CO_2_ transport on O_2_ transport.

**Figure 5:**
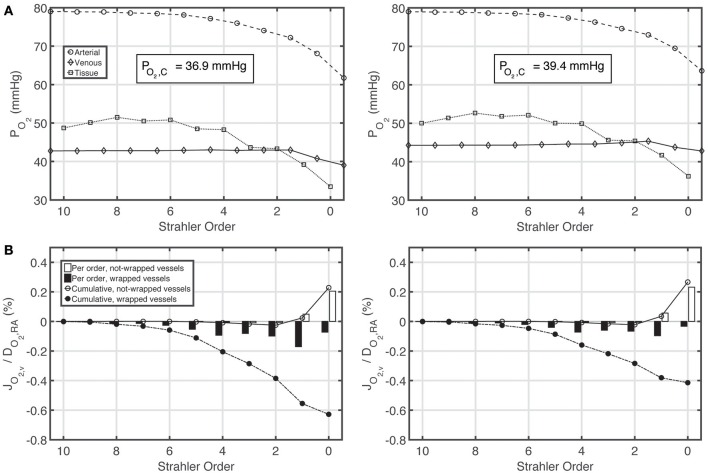
**Comparison of results for the base case with constant P_**50**_ (left) and variable P_**50**_ dependent on the local P_**CO**_**2**__ and pH based on ***Condition 2*** (right). (A)** Arterial, venous and tissue partial pressure of oxygen (P_O_2__) profiles, and average cortical oxygen partial pressure, P_O_2_, C_, **(B)** Oxygen flux across vein walls, J_O_2, v__, reported as percentage of total renal oxygen delivery, D_O_2_, RA_. Cumulative values as well as values for each individual order are shown. Positive flux represents flux from the vessel into the tissue, whereas negative flux denotes the opposite, i.e., negative J_O_2_, v_ denotes oxygen shunted to the venous tree.

Figures 5A,B show P_O_2__ profiles in arteries, veins and tissue, as well as oxygen fluxes between vein walls and tissue. The P_O_2__ profiles for the constant and variable P_50_ cases are very similar, with a slight overall increase in P_O_2__ for the variable P_50_ case due to higher P_50_ compared to the constant P_50_ case. For the constant P_50_ case, cumulatively 0.6% of the total renal oxygen delivery is shunted from the arteries to the veins along wrapped artery-vein pairs, whereas 0.2% of the total renal oxygen delivery is supplied to the tissue by the veins along not-wrapped artery-vein pairs. Hence, for this case, the total preglomerular AV oxygen shunting is 0.4% of the total renal oxygen delivery. Note that these results are slightly different from the ones given in our previous study (Olgac and Kurtcuoglu, 2015b). This is due to the fact that we used P_50_ = 34 mmHg there compared to P_50_ = 26.8 mmHg in the current study.

Under variable P_50_ conditions, oxygen shunting along the wrapped vessels decreases, with overall preglomerular AV oxygen shunting reducing to 0.15% of the total renal oxygen delivery. Hence, CO_2_ effects do not promote but rather decrease preglomerular AV O_2_ shunting. This is because the increase in acidity (and hence the decrease in the affinity of hemoglobin to oxygen) is more pronounced on the venous compared to the arterial side.

### Effects of buffering capacity

**Figure 6:**
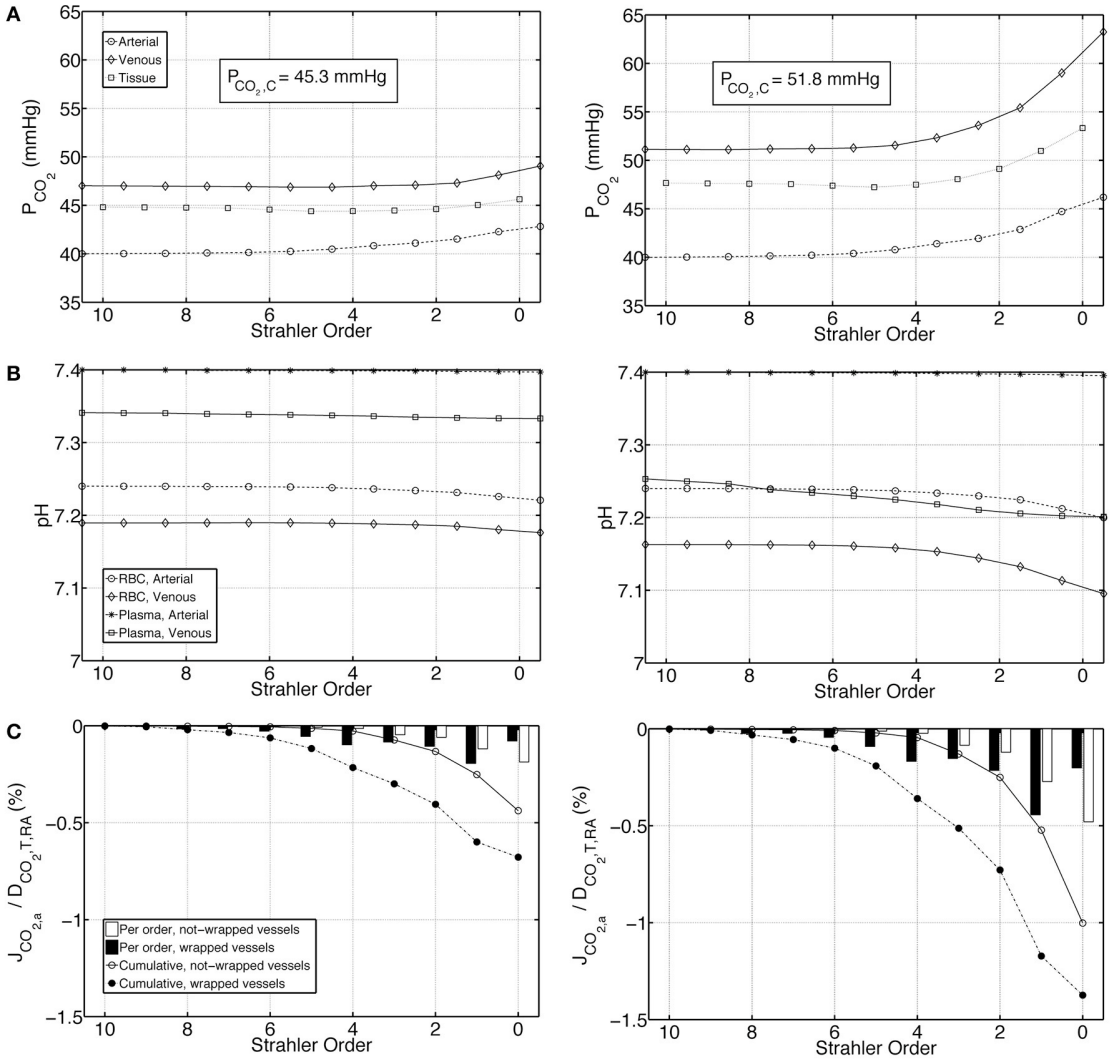
**Comparison of results for a case with tenfold decreased plasma and RBC buffering capacity under ***Condition 1*** (left) and ***Condition 2*** (right), (A)** Arterial, venous and tissue partial pressure of carbon dioxide (P_CO_2__) profiles, **(B)** Arterial and venous plasma and RBC pH profiles, **(C)** Carbon dioxide flux across artery walls, J_CO_2, a__, reported as percentage of total renal carbon dioxide delivery, D_CO_2_, T, RA_. Cumulative values as well as values for each individual order are shown. Positive flux represents flux from the vessel into the tissue, whereas negative flux denotes the opposite, i.e., negative J_CO_2_, a_ denotes carbon dioxide shunted to the arterial tree.

To test the influence of buffering capacity, we performed calculations on a modified base case with a 10 fold decrease in plasma and RBC buffering capacities. Figures 6A–C represent, on the left panel under *Condition 1* and on the right panel under *Condition 2*, P_CO_2__, pH and carbon dioxide flux profiles for this modified case. Compared to the base case results presented in Figure 4, under both *Conditions 1 & 2*, the increase in P_CO_2__ and decrease in pH are more pronounced when the buffering capacity is decreased. P_CO_2__ increases to 42.8 and 46.2 mmHg at the outlet of the afferent arteriole (order 0) under *Conditions 1 & 2*, respectively. On the venous return, under *Condition 1*, P_CO_2__ = 49.1 mmHg, whereas under *Condition 2*, P_CO_2__ = 63.2 mmHg. Furthermore, pH profiles vary more compared to the rather flat profiles in Figure 4. On the venous return, plasma and RBC pH are 7.30 and 7.18, respectively, under *Condition 1*, compared to 7.20 and 7.10, respectively, under *Condition 2*. VA CO_2_ shunting is also increased due to the increased P_CO_2__ gradient between the venous and the arterial side, with 1.2 and 2.4% of the total renal CO_2_ delivery shunted from the preglomerular veins to the arteries under *Conditions 1 & 2*, respectively.

## Discussion

We have made the following key observations: 1 Increase in acidity in the preglomerular vasculature compared to systemic arterial blood is marginal. 2 CO_2_ effects do not promote preglomerular arterial-to-venous O_2_ shunting but rather impair it. In the following we will discuss these observations.

### Marginal increase in acidity in the preglomerular vasculature

We calculated the P_CO_2__ at the outlet of the afferent arteriole and on the venous return to be in the range of 40.8–42.3 mmHg and 43.5–52.8 mmHg, respectively, for a renal arterial P_CO_2__ of 40 mmHg. Plasma and RBC pH on the venous return decrease to somewhere between 7.28–7.39 and 7.21–7.23, respectively, compared to their systemic arterial values of 7.4 and 7.24, respectively. Taken together, we conclude that the increase in acidity in the preglomerular vasculature is not substantial. The main reason for this is the high buffering capacity of blood. When we lowered the buffering capacity in our model, acidity increased substantially. It should be noted that there may be more pronounced increase in acidity in the postglomerular vasculature, along the peritubular capillary network and/or the vasa recta. Our current model does not include these parts.

Previous modeling studies by Bidani et al. (1984) and Atherton et al. (1988) were based on the proximal tubular P_CO_2__ of 65 mmHg measured by DuBose et al. (1979). They indicated that substantial venous-to-arterial CO_2_ shunting would be necessary to preserve this P_CO_2__ of 65 mmHg in the renal cortex. Here we calculated average cortical P_CO_2__, P_CO_2_, C_, to be between 42.0 and 46.0 mmHg. We further calculated the shunting of CO_2_ from the veins to the arteries to be approximately between 0.5 and 1.4% of the total renal carbon dioxide delivery. We conclude that just as the increase in acidity, the venous-to-arterial CO_2_ shunting in the preglomerular vasculature is also only marginal.

### CO_2_ effects impair preglomerular AV O_2_ shunting

We observed that when calculations are based on a variable P_50_ that is dependent on the local P_CO_2__ and pH, the AV O_2_ shunting decreases. This is mainly because the increase in acidity is higher on the venous side, which leads to a lower affinity of hemoglobin to oxygen compared to the arterial side. This lower affinity on the venous side makes it slightly harder for the oxygen to bind to hemoglobin, diminishing the oxygen transfer from the tissue into the veins, hence decreasing AV O_2_ shunting. Therefore, our model does not support the hypothesis initially proposed by Schurek et al. (1990) that renal CO_2_ trapping through a reverse CO_2_ shunting mechanism may enhance AV O_2_ shunting through the Bohr effect. The current model thus also confirms our previous findings that preglomerular AV O_2_ shunting is marginal, and that if substantial renal oxygen shunting exists, it should be along the post-glomerular vasculature, i.e., the peritubular capillary network and/or the vasa recta (Olgac and Kurtcuoglu, 2015a,b).

### Limitations of the model

The main limitation of the model is that the distribution of the different forms of CO_2_ in the venous return is unknown. To address this, two different conditions representing extreme cases were employed and ranges of values reported. The actual values representing the real state are expected to lie within the given ranges. More accurate calculations would require that CO_2_ transport dynamics in the peritubular capillary network be taken into account, which would necessitate explicit treatment of the postglomerular vasculature and the tubular system. Modeling bicarbonate reabsorption in this domain would yield an estimate of what fraction of the carbon dioxide produced in the tubular cells reaches the capillaries in bicarbonate and CO_2_ form, respectively. This fraction is unknown in the current model. Nevertheless, the two conditions employed on the venous return provide solid boundaries for the ranges of P_CO_2__ and pH in the preglomerular vasculature, and the conclusions reached in this study are valid for both conditions.

A further limitation of this computational study is produced by the fact that there are no comparable experimental studies of renal carbon dioxide transport which could be used for validation. The experimental P_CO_2__ and pH measurements referred to in the *Introduction* section have been performed on tubules and stellate vessels, and can thus not be compared with our results. Quantitative cortical tissue and afferent arteriole P_CO_2__ and pH values have, to our knowledge, not been published. To test the robustness of our conclusions, we performed sensitivity analyses in which we altered renal artery inlet boundary conditions.

First, we established alternative chemical equilibria at the renal artery inlet corresponding to renal artery P_CO_2__ values of 35 mmHg and 45 mmHg, respectively. This accounts for possible variation in renal artery P_CO_2__ in the physiologic range. We calculated maximum P_CO_2__ at the outlet of the afferent arteriole and on the venous return to be 37.1 and 47.1 mmHg, respectively, for renal artery P_CO_2__ of 35 mmHg, and 47.6 and 58.5 mmHg, respectively, for renal artery P_CO_2__ of 45 mmHg (Supplementary Figure 1, Supplementary Table 1). These extreme cases show relative increases in P_CO_2__ in the preglomerular vasculature with respect to renal artery P_CO_2__ that are similar to the base case. Plasma and RBC pH on the venous return decrease to minima of 7.32 and 7.22, respectively, for renal artery P_CO_2__ of 35 mmHg, plasma pH of 7.45 and RBC pH of 7.25 (Supplementary Figure 1, Supplementary Table 1). For the other extreme case with renal artery P_CO_2__ of 45 mmHg, plasma pH of 7.35 and RBC pH of 7.23, we calculated minimum plasma and RBC pH on the venous return to be 7.24 and 7.20, respectively (Supplementary Figure 1, Supplementary Table 1). These relative decreases in pH in the preglomerular vasculature with respect to renal artery pH in the extreme cases are similar to that in the base case. Furthermore, oxygen transport calculations in these extreme cases under variable P_50_ conditions show impaired AV oxygen shunting compared to under constant P_50_ conditions, just as it was observed in the base case (Supplementary Figures 2, 3). There, under variable P_50_ conditions, overall preglomerular oxygen shunting reduced to 0.15% of the total renal oxygen delivery, compared to 0.40% under constant P_50_ conditions. In the extreme cases, this reduction was to 0.20 and 0.10% of the total renal oxygen delivery for renal arterial P_CO_2__ of 35 mmHg and 45 mmHg, respectively (Supplementary Table 2).

In a second analysis, we altered the renal blood flow rate (RBF) by 30% in either direction. With a 30% decrease in RBF, the increase in acidity in the preglomerular vasculature became more pronounced. We calculated for this case maximum P_CO_2__ at the outlet of the afferent arteriole and on the venous return to be 44.1 and 59.6 mmHg, respectively (Supplementary Figure 4, Supplementary Table 3). Plasma and RBC pH on the venous return decrease to minima of 7.23 and 7.20, respectively. Conversely, with a 30% increase in RBF, the increase in acidity in the preglomerular vasculature became less pronounced with maximum P_CO_2__ of 41.6 and 49.5 mmHg at the outlet of the afferent arteriole and on the venous return, respectively, and minimum plasma and RBC pH on the venous return of 7.31 and 7.22, respectively (Supplementary Figure 4, Supplementary Table 3). In these extreme cases, AV O_2_ shunting reduced from 0.81% (under constant P_50_ conditions) to 0.32% (under variable P_50_ conditions) of the total renal oxygen delivery (with 30% decrease in RBF) and from 0.21% (under constant P_50_ conditions) to 0.05% (under variable P_50_ conditions) of the total renal oxygen delivery (with 30% increase in RBF) (Supplementary Figures 5, 6, Supplementary Table 4). In comparison, in the base case, AV O_2_ shunting reduced from 0.40% (under constant P_50_ conditions) to 0.15% (under variable P_50_ conditions) of the total renal oxygen delivery (Supplementary Table 4).

We conclude that our first main observation, namely that increase in acidity in the preglomerular vasculature compared to systemic arterial blood is marginal, is robust unless RBF is substantially reduced. Our second main observation, i.e., that CO_2_ effects do not promote preglomerular arterial-to-venous O_2_ shunting but rather impair it, is robust for all investigated nominal and extreme conditions.

## Conclusions

Our model suggests that under normal physiologic conditions, the increase in acidity in the preglomerular vasculature compared to systemic arterial blood is marginal, and that venous-to-arterial shunting of carbon dioxide does not promote, but rather impairs preglomerular arterial-to-venous oxygen shunting.

## Author contributions

UO and VK designed this research; UO and VK developed the mathematical model; UO implemented the computational model and performed the computations; UO and VK interpreted results of the computations; UO prepared figures; UO drafted the manuscript; UO and VK edited and revised the manuscript; UO and VK approved final version of the manuscript.

## Funding

The financial support of the Swiss National Center of Competence in Research on Control of Homeostasis by the Kidney (NCCR Kindey.CH) is kindly acknowledged.

### Conflict of interest statement

The authors declare that the research was conducted in the absence of any commercial or financial relationships that could be construed as a potential conflict of interest.

## Nomenclature

AV  Arterial-to-venous

$\hat{\text{A}}$ Carbonic anhydrase activity factor

BC  Boundary condition

C  Concentration

D^P^  Diffusion coefficient in plasma

D^T^  Diffusion coefficient in tissue

D  Delivery

DPG  2,3-Diphosphoglycerate

e  Relative error

f_water_  Water fraction of total RBC volume

h(r)  Radial hematocrit profile

H_c_  Capillary hematocrit

J  Flux

K  Permeability

K_A_  Equilibrium association rate constant

K′  Apparent dissociation constant for H_2_CO_3_

K_1_  First dissociation constant for H_2_CO_3_

K_DPG_  Association constant for DPG and hemoglobin

k  Number of vessels

k_trans_  Translocation rate constant

k_u_ CO_2_  hydration reaction rate

k_v_ H_2_CO_3_  dehydration rate constant

l  Length of vessel

LS  Lumen separation

$\dot{\text{M}}$  Local consumption/production rate

${\dot{\text{M}}}_{\text{c}}$  Capillary source/sink

N_A_  Avogadro number

n  Empirical constant in Hill equation

P_50_  Half saturation oxygen partial pressure

P  Partial pressure

r  Radial location

R  Reaction rate

R_a_  Artery radius

R_v_  Vein radius

RBC  Red blood cell

R_RBC_  Radius of RBC disk

RBF  Renal blood flow rate

RQ  Respiratory quotient

S_O_2__  Saturation of hemoglobin with oxygen

t_RBC_  Maximum half thickness of RBC

T_tot_  Total number of anion transporters on RBC membrane

u  Oxygen advection velocity in capillaries

u(r)  Radial velocity profile in RBC-rich region

u′(r)  Radial velocity profile in RBC-free region

$\dot{\text{V}}$  Consumption/production rate

VA  Venous-to-arterial

WKY  Wistar Kyoto rat

z  Axial location

α  Solubility coefficient

β  Buffering capacity

δ  Thickness of RBC-free region

λ_α_  Association constant for CO_2_ binding to the α-chain of hemoglobin

λ_β_  Association constant for CO_2_ binding to the β-chain of hemoglobin

φ  Fractional capillary volume

Superscripts and subscripts

a  Arterial

AT  Anion transporter

c  Capillary

C  Cortical

Cl^−^  Chloride ion

CO_2_  Carbon dioxide

H^+^  Hydrogen ion

HbO_2_  Heme groups bound to oxygen

HbCO_2_  Hemoglobin carbamate

HbCO_2, T_  Total hemoglobin bound CO2

O_2_HbCO_2_  Oxyhemoglobin carbamate

HbT  Total heme groups

${\text{HCO}}_{3}^{-}$  Bicarbonate ion

i  Representative level

M  Medullary

nw  Not-wrapped

O_2_  Oxygen

P  Plasma

RA  Renal artery

RBC  Red blood cell

S  Standard physiological

T  Tissue

v  Venous

w  wrapped
