# Supplementary material for: The Bohr Effect Is Not a Likely Promoter of Renal Preglomerular Oxygen Shunting
Source: Front Physiol. 2016 Oct 27;7:482. doi: 10.3389/fphys.2016.00482 (PMC5081373; doi:10.3389/fphys.2016.00482)
Supplement: Supplementary file 1 [file Table1.DOCX]

**Table 1.** Sensitivity analysis with respect to renal arterial inlet PCO2: Comparison of maximum $P_{CO_{2}}$ at the outlet of the afferent arteriole and on the venous return and minimum plasma and RBC pH on the venous return

|  | Maximum $P_{CO_{2}}$ at the outlet of the afferent arteriole | Maximum $P_{CO_{2}}$ on the venous return | | Minimum plasma pH on the venous return | | Minimum RBC pH on the venous return | |
| --- | --- | --- | --- | --- | --- | --- | --- |
| $P_{\mathrm{CO}_{2},RA}$ = 35 mmHg,  $\mathrm{pH}_{P,RA}$ = 7.45, $\mathrm{pH}_{RBC,RA}$ = 7.25 | 37.1 | 47.1 | 7.32 | | 7.22 | |  |
| $P_{\mathrm{CO}_{2},RA}$ = 40 mmHg,  $\mathrm{pH}_{P,RA}$ = 7.4, $\mathrm{pH}_{RBC,RA}$ = 7.24 | 42.3 | 52.8 | 7.28 | | 7.21 | |  |
| $P_{\mathrm{CO}_{2},RA}$ = 45 mmHg,  $\mathrm{pH}_{P,RA}$ = 7.35, $\mathrm{pH}_{RBC,RA}$ = 7.23 | 47.6 | 58.5 | 7.24 | | 7.20 | |  |
