# Supplementary material for: The Bohr Effect Is Not a Likely Promoter of Renal Preglomerular Oxygen Shunting
Source: Front Physiol. 2016 Oct 27;7:482. doi: 10.3389/fphys.2016.00482 (PMC5081373; doi:10.3389/fphys.2016.00482)
Supplement: Supplementary file 2 [file Table2.DOCX]

**Table 2.** Sensitivity analysis with respect to renal arterial inlet PCO2: Comparison of oxygen flux across vein walls, $J_{O_{2,v}}$, reported as percentage of total renal oxygen delivery, $D_{O_{2},RA}$.

|  | $J_{O_{2,v}}/D_{O_{2},RA}$, % | | |
| --- | --- | --- | --- |
|  | Constant P50 | Variable P50 | |
| $P_{\mathrm{CO}_{2},RA}$ = 35 mmHg | -0.40 | -0.20 |  |
| $P_{\mathrm{CO}_{2},RA}$ = 40 mmHg | -0.40 | -0.15 |  |
| $P_{\mathrm{CO}_{2},RA}$ = 45 mmHg | -0.40 | -0.10 |  |
