# Supplementary material for: The Bohr Effect Is Not a Likely Promoter of Renal Preglomerular Oxygen Shunting
Source: Front Physiol. 2016 Oct 27;7:482. doi: 10.3389/fphys.2016.00482 (PMC5081373; doi:10.3389/fphys.2016.00482)
Supplement: Supplementary file 3 [file Table3.DOCX]

**Table 3.** Sensitivity analysis with respect to renal blood flowrate (RBF): Comparison of maximum $P_{CO_{2}}$ at the outlet of the afferent arteriole and on the venous return and minimum plasma and RBC pH on the venous return

|  | Maximum $P_{CO_{2}}$ at the outlet of the afferent arteriole | Maximum $P_{CO_{2}}$ on the venous return | | Minimum plasma pH on the venous return | | Minimum RBC pH on the venous return | |
| --- | --- | --- | --- | --- | --- | --- | --- |
| 30% decrease in RBF | 44.1 | 59.6 | 7.23 | | 7.20 | |  |
| Base case | 42.3 | 52.8 | 7.28 | | 7.21 | |  |
| 30% increase in RBF | 41.6 | 49.5 | 7.31 | | 7.22 | |  |
