# Supplementary material for: The Bohr Effect Is Not a Likely Promoter of Renal Preglomerular Oxygen Shunting
Source: Front Physiol. 2016 Oct 27;7:482. doi: 10.3389/fphys.2016.00482 (PMC5081373; doi:10.3389/fphys.2016.00482)
Supplement: Supplementary file 4 [file Table4.DOCX]

**Table 4.** Sensitivity analysis with respect to renal blood flowrate (RBF): Comparison of oxygen flux across vein walls, $J_{O_{2,v}}$, reported as percentage of total renal oxygen delivery, $D_{O_{2},RA}$.

|  | $J_{O_{2,v}}/D_{O_{2},RA}$, % | | |
| --- | --- | --- | --- |
|  | Constant P50 | Variable P50 | |
| 30% decrease in RBF | -0.81 | -0.32 |  |
| Base case | -0.40 | -0.15 |  |
| 30% increase in RBF | -0.21 | -0.05 |  |
