# Supplementary material for: The Bohr Effect Is Not a Likely Promoter of Renal Preglomerular Oxygen Shunting
Source: Front Physiol. 2016 Oct 27;7:482. doi: 10.3389/fphys.2016.00482 (PMC5081373; doi:10.3389/fphys.2016.00482)
Supplement: Supplementary file 5 [file DataSheet1.docx]

# Data Sheet

# Mathematical formulation

## Oxygen transport model

The intravascular oxygen transport is based on (Olgac and Kurtcuoglu, 2015a) and considers axial convection of oxygen with plasma and RBCs, as well as radial diffusion. Inside the RBCs, oxygen binds to heme groups in hemoglobin, which can be described by a one-step reaction,

|  | $\mathrm{Hb}O_{2}\leftrightarrow Hb+O_{2}.$ | **A.1** |
| --- | --- | --- |

The governing equations for oxygen transport in the RBC-rich region can be written as

|  | $u_{\mathrm{RBC}}\left( r \right)\frac{\partial C_{O_{2}}^{\mathrm{RBC}}}{\partial z}=-J_{O_{2}}^{\mathrm{RBC}}\left[ \frac{s}{v} \right]_{\mathrm{RBC}}+R_{O_{2}},$ | **A.2** |
| --- | --- | --- |
|  | $u_{\mathrm{RBC}}\left( r \right)\frac{\partial C_{\mathrm{HbO}_{2}}^{\mathrm{RBC}}}{\partial z}=-R_{O_{2}},$ | **A.3** |
|  | ${(1-h(r))u}_{P}\left( r \right)\frac{\partial C_{O_{2}}^{P}}{\partial z}=\frac{D}{r}\frac{\partial}{\partial r}\left( r\frac{\partial C_{O_{2}}^{P}}{\partial r} \right)+J_{O_{2}}^{\mathrm{RBC}}h\left( r \right)\left[ \frac{s}{v} \right]_{\mathrm{RBC}},$ | **A.4** |

where $J_{O_{2}}^{\mathrm{RBC}}$ is the flux across the RBC membrane, $\left[ \frac{s}{v} \right]_{\mathrm{RBC}}$ is the surface to volume ratio of an RBC and $R_{O_{2}}$ is the net amount of oxygen released in the reaction given in **A.1.** Combining Eqs. **A.2-4** and assuming a continuous oxygen tension across the RBC wall,

|  | $\left[ {{(1-h(r))u}_{P}\left( r \right)+\frac{\alpha_{\mathrm{RBC}}}{\alpha_{P}}h(r)u}_{\mathrm{RBC}}\left( r \right) \right]\frac{\partial C_{O_{2}}^{\mathrm{RBC}}}{\partial z}+\frac{\alpha_{\mathrm{RBC}}}{\alpha_{P}}h\left( r \right)u_{\mathrm{RBC}}\left( r \right)\frac{\partial C_{\mathrm{HbO}_{2}}^{\mathrm{RBC}}}{\partial z}=\frac{D}{r}\frac{\partial}{\partial r}\left( r\frac{\partial C_{O_{2}}^{P}}{\partial r} \right).$ | **A.5** |
| --- | --- | --- |

We then assume that the reaction given in **A.1** is in chemical equilibrium and that the saturation of heme groups with oxygen, $S_{O_{2}}$, is represented by the Hill equation (Clark et al., 1985)

|  | $S_{O_{2}}=\frac{C_{\mathrm{HbO}_{2}}}{C_{\mathrm{HbT}}}=\frac{{({C_{O_{2}}^{\mathrm{RBC}}}/{C_{50}})}^{n}}{1+{({C_{O_{2}}^{\mathrm{RBC}}}/{C_{50}})}^{n}} ,$ | **A.6** |
| --- | --- | --- |

where $C_{50}$ and $n$ are the half saturation oxygen concentration and an empirical constant, respectively. Hence,

|  | $\frac{\partial C_{\mathrm{HbO}_{2}}^{\mathrm{RBC}}}{\partial z}=C_{\mathrm{HbT}}\left[ \frac{dS_{O_{2}}}{dC_{O_{2}}^{\mathrm{RBC}}}\frac{\partial C_{O_{2}}^{\mathrm{RBC}}}{\partial z}-\frac{C_{O_{2}}^{\mathrm{RBC}}}{C_{50}}\frac{dS_{O_{2}}}{dC_{O_{2}}^{\mathrm{RBC}}}\frac{\partial C_{50}}{\partial z} \right],$ | **A.7** |
| --- | --- | --- |

where

|  | $\frac{dS_{O_{2}}}{dC_{O_{2}}^{\mathrm{RBC}}}=\frac{n{({C_{O_{2}}^{\mathrm{RBC}}}/{C_{50}})}^{n}}{{C_{O_{2}}^{\mathrm{RBC}}(1+\left( {C_{O_{2}}^{\mathrm{RBC}}}/{C_{50}} \right)^{n})}^{2}}.$ | **A.8** |
| --- | --- | --- |

Substituting Eq. **A.7** into Eq. **A.5**:

|  | $\left[ \left( 1-h\left( r \right) \right)u_{P}\left( r \right)+\frac{\alpha_{\mathrm{RBC}}}{\alpha_{P}}h\left( r \right)u_{\mathrm{RBC}}\left( r \right)\left( 1+C_{\mathrm{HbT}}\frac{dS_{O_{2}}}{dC_{O_{2}}^{\mathrm{RBC}}} \right) \right]\frac{\partial C_{O_{2}}^{\mathrm{RBC}}}{\partial z}-\frac{\alpha_{\mathrm{RBC}}}{\alpha_{P}}h\left( r \right)u_{\mathrm{RBC}}\left( r \right)C_{\mathrm{HbT}}\frac{C_{O_{2}}^{\mathrm{RBC}}}{C_{50}}\frac{dS_{O_{2}}}{dC_{O_{2}}^{\mathrm{RBC}}}\frac{\partial C_{50}}{\partial z}=\frac{D}{r}\frac{\partial}{\partial r}\left( r\frac{\partial C_{O_{2}}^{\mathrm{RBC}}}{\partial r} \right).$ | **A.9** |
| --- | --- | --- |

Equation **1** can be obtained from Eq. **A.9** by inserting $C_{O_{2}}^{\mathrm{RBC}}=\alpha_{\mathrm{RBC}}P_{O_{2}}$ and $C_{50}=\alpha_{\mathrm{RBC}}P_{50}$.

## Carbon dioxide transport model

The carbon dioxide transport model considers axial convection of carbon dioxide, bicarbonate ion, hydrogen ion and chloride ion with plasma and RBCs, as well as radial diffusion of these species in plasma. Carbon dioxide also diffuses through the tissue, whereas the vessel walls are assumed impermeable to the remaining species. In plasma and RBC, carbon dioxide combines with water to form carbonic acid through the reversible hydration/dehydration reaction

|  | $CO_{2}+H_{2}O\leftrightarrow H_{2}CO_{3},$ | **A.10** |
| --- | --- | --- |

for which k_u_ and k_v_ are the forward and backward reaction rates, respectively. This reaction is slow in plasma, whereas due to the presence of the enzyme carbonic anhydrase, it is very fast inside RBCs. Both in plasma and RBC, carbonic acid instantly dissociates into bicarbonate and hydrogen ions with a first acid dissociation constant of K_1_:

|  | $H_{2}CO_{3}\leftrightarrow\mathrm{HCO}_{3}^{-}+H^{+}.$ | **A.11** |
| --- | --- | --- |

Hence, the rate of carbon dioxide hydration (or bicarbonate formation, $R_{\mathrm{HCO}_{3}^{-}}$) can be expressed as

|  | $-\frac{dC_{CO_{2}}}{\mathrm{dt}}=R_{\mathrm{HCO}_{3}^{-}}=\hat{A}\left( k_{u}C_{CO_{2}}-\frac{k_{v}}{K_{1}}C_{H^{+}}C_{\mathrm{HCO}_{3}^{-}} \right),$ | **A.12** |
| --- | --- | --- |

where $\hat{A}$ is the carbonic anhydrase activity factor, which is taken as unity in plasma due to the non-existence of carbonic anhydrase. Consequently, the reaction rate in plasma can be expressed as

|  | $R_{\mathrm{HCO}_{3}^{-}}^{P}=k_{u}^{P}C_{CO_{2}}^{P}-\frac{k_{v}^{P}}{K_{1}^{P}}C_{H^{+}}^{P}C_{\mathrm{HCO}_{3}^{-}}^{P}.$ | **A.13** |
| --- | --- | --- |

Since $\hat{A}^{\mathrm{RBC}}$ is 6500 – 15000 (Huang, 1991; Huang and Hellums, 1994), the hydration reaction inside RBC is very fast. Therefore, we assume that the hydration/dehydration reaction to be in equilibrium in RBC:

|  | $C_{H^{+}}^{\mathrm{RBC}}C_{\mathrm{HCO}_{3}^{-}}^{\mathrm{RBC}}=K^{',RBC}f_{\mathrm{water}}C_{CO_{2}}^{\mathrm{RBC}},$ | **A.14** |
| --- | --- | --- |

where $K^{',RBC}$ is the apparent first dissociation constant for H_2_CO_3_ in RBC, defined as $K^{',RBC}=K_{1}^{\mathrm{RBC}}({k_{u}^{\mathrm{RBC}}}/{k_{v}^{\mathrm{RBC}}})$, and f_water_ is the water fraction of total RBC volume.

Hydrogen ions inside RBCs are buffered by hemoglobin and over the physiological range of pH, the non-$\mathrm{HCO}_{3}^{-}$ titration curve is linear (Davenport, 1958; Boron and Boulpaep, 2012):

|  | $C_{\mathrm{HCO}_{3}^{-}}^{\mathrm{RBC}}=-\beta_{\mathrm{RBC}}\mathrm{pH}_{\mathrm{RBC}}+b,$ | **A.15** |
| --- | --- | --- |

where $pH=-logC_{H^{+}}$. Equation **A.15** is combined with Eq. **A.14** to result in

|  | ${(\beta}_{\mathrm{RBC}}\log C_{H^{+}}^{\mathrm{RBC}}+b)C_{H^{+}}^{\mathrm{RBC}}=K^{',RBC}f_{\mathrm{water}}C_{\mathrm{CO}_{2}}^{\mathrm{RBC}}.$ | **A.16** |
| --- | --- | --- |

Equations **A.15** and **A.16** are equivalent to Eqs. **8** and **9**, respectively, and solved to obtain bicarbonate and hydrogen ion concentration in RBCs. The governing equations for the remaining species of carbon dioxide transport are based on the model presented by Huang and Hellums (Huang, 1991; Huang and Hellums, 1994): Governing equations for the transport of chloride ion in RBCs and bicarbonate, hydrogen and chloride ions in plasma (Eqs. **10 - 13**) are directly adopted (Huang, 1991; Huang and Hellums, 1994), whereas the governing equation for carbon dioxide partial pressure (Eq. **7**) is slightly modified after following derivation: the concentration of carbon dioxide in plasma and in RBC follows (Huang, 1991; Huang and Hellums, 1994)

|  | $u_{\mathrm{RBC}}\left( r \right)\frac{\partial C_{\mathrm{CO}_{2}}^{\mathrm{RBC}}}{\partial z}=J_{\mathrm{CO}_{2}}^{\mathrm{RBC}}\left[ \frac{s}{v} \right]_{\mathrm{RBC}}-R_{\mathrm{HCO}_{3}^{-}}^{\mathrm{RBC}}-R_{\mathrm{HbCO}_{2}}^{\mathrm{RBC}}-R_{O_{2}\mathrm{HbCO}_{2}}^{\mathrm{RBC}},$ | **A.17**(a) |
| --- | --- | --- |
|  | $\left( 1-h\left( r \right) \right)u_{P}\left( r \right)\frac{\partial C_{\mathrm{CO}_{2}}^{P}}{\partial z}=\frac{D_{\mathrm{CO}_{2}}^{P}}{r}\frac{\partial}{\partial r}\left( r\frac{\partial C_{\mathrm{CO}_{2}}^{P}}{\partial r} \right)-h\left( r \right)J_{\mathrm{CO}_{2}}^{\mathrm{RBC}}\left[ \frac{s}{v} \right]_{\mathrm{RBC}}-\left( 1-h\left( r \right) \right)R_{\mathrm{HCO}_{3}^{-}}^{P},$ | **A.17**(b) |

where $J_{\mathrm{CO}_{2}}^{\mathrm{RBC}}$ is the carbon dioxide flux across the RBC membrane, $\left[ \frac{s}{v} \right]_{\mathrm{RBC}}$ is the surface area to volume ratio of an RBC, and $R_{\mathrm{HCO}_{3}^{-}}^{\mathrm{RBC}}$, $R_{\mathrm{HbCO}_{2}}^{\mathrm{RBC}}$ and $R_{O_{2}\mathrm{HbCO}_{2}}^{\mathrm{RBC}}$ are the rate of bicarbonate ion, hemoglobin carbamate and oxyhemoglobin carbamate formation, respectively. We neglect the resistance of the RBC membrane, as well as the resistances associated with the intra- and extracellular boundary layers. Hence, we assume a continuous carbon dioxide tension across the RBC wall. Consequently, using $C_{\mathrm{CO}_{2}}^{P}=(\alpha_{\mathrm{CO}_{2}}^{P}/\alpha_{\mathrm{CO}_{2}}^{\mathrm{RBC}})C_{CO_{2}}^{\mathrm{RBC}}$, Eqs. **A.17**(a) and **A.17**(b) can be combined to obtain an equation for the carbon dioxide concentration in RBCs in the RBC-rich region, $C_{CO_{2}}^{\mathrm{RBC}}$:

|  | $\left[ h\left( r \right)u_{\mathrm{RBC}}\left( r \right)+\frac{\alpha_{\mathrm{CO}_{2}}^{P}}{\alpha_{\mathrm{CO}_{2}}^{\mathrm{RBC}}}\left( 1-h\left( r \right) \right)u_{P}\left( r \right) \right]\frac{\partial C_{\mathrm{CO}_{2}}^{\mathrm{RBC}}}{\partial z}=(\frac{\alpha_{\mathrm{CO}_{2}}^{P}}{\alpha_{\mathrm{CO}_{2}}^{\mathrm{RBC}}})\frac{D_{\mathrm{CO}_{2}}^{P}}{r}\frac{\partial}{\partial r}\left( r\frac{\partial C_{\mathrm{CO}_{2}}^{\mathrm{RBC}}}{\partial r} \right)$  $-{h\left( r \right)R}_{\mathrm{HCO}_{3}^{-}}^{\mathrm{RBC}}-\left( 1-h\left( r \right) \right)R_{\mathrm{HCO}_{3}^{-}}^{P}-{h\left( r \right)R}_{\mathrm{HbCO}_{2}}^{\mathrm{RBC}}-h(r)R_{O_{2}\mathrm{HbCO}_{2}}^{\mathrm{RBC}},$ | **A.18** |
| --- | --- | --- |

The reaction terms in RBC in Eq. **A.18** are obtained from the governing equation of the respective species (Huang, 1991; Huang and Hellums, 1994):

|  | $u_{\mathrm{RBC}}\left( r \right)\frac{\partial C_{\mathrm{HbCO}_{2}}}{\partial z}=R_{\mathrm{HbCO}_{2}}^{\mathrm{RBC}},$ | **A.19**(a) |
| --- | --- | --- |
|  | $u_{\mathrm{RBC}}\left( r \right)\frac{\partial C_{O_{2}\mathrm{HbCO}_{2}}}{\partial z}=R_{O_{2}\mathrm{HbCO}_{2}}^{\mathrm{RBC}},$ | **A.19**(b) |
|  | $u_{\mathrm{RBC}}\left( r \right)\frac{\partial C_{\mathrm{HCO}_{3}^{-}}^{\mathrm{RBC}}}{\partial z}=J_{\mathrm{HCO}_{3}^{-}}\left[ \frac{s}{v} \right]_{\mathrm{RBC}}+R_{\mathrm{HCO}_{3}^{-}}^{\mathrm{RBC}}.$ | **A.19**(c) |

Substituting the reaction terms from Eq. **A.19** into Eq. **A.18** and defining total hemoglobin bound CO_2_ as $\mathrm{HbCO}_{2,T}=\mathrm{HbCO}_{2}+O_{2}\mathrm{HbCO}_{2}$, Eq. **A.18** can be written as

|  | $\left[ h\left( r \right)u_{\mathrm{RBC}}\left( r \right)+\frac{\alpha_{\mathrm{CO}_{2}}^{P}}{\alpha_{\mathrm{CO}_{2}}^{\mathrm{RBC}}}\left( 1-h\left( r \right) \right)u_{P}\left( r \right) \right]\frac{\partial C_{\mathrm{CO}_{2}}^{\mathrm{RBC}}}{\partial z}=(\frac{\alpha_{\mathrm{CO}_{2}}^{P}}{\alpha_{\mathrm{CO}_{2}}^{\mathrm{RBC}}})\frac{D_{\mathrm{CO}_{2}}^{P}}{r}\frac{\partial}{\partial r}\left( r\frac{\partial C_{\mathrm{CO}_{2}}^{\mathrm{RBC}}}{\partial r} \right)$  $-{h\left( r \right)u}_{\mathrm{RBC}}\left( r \right)\frac{\partial C_{\mathrm{HCO}_{3}^{-}}^{\mathrm{RBC}}}{\partial z}-{h\left( r \right)u}_{\mathrm{RBC}}\left( r \right)\frac{\partial C_{\mathrm{HbCO}_{2,T}}}{\partial z}$  $-\left( 1-h\left( r \right) \right)R_{\mathrm{HCO}_{3}^{-}}^{P}+h\left( r \right)J_{\mathrm{HCO}_{3}^{-}}\left[ \frac{s}{v} \right]_{\mathrm{RBC}}.$ | **A.20** |
| --- | --- | --- |

With the application of the chain rule to the axial derivatives on the right hand side, Eq. **A.20** is reorganized as follows

|  | $\left[ \frac{\alpha_{\mathrm{CO}_{2}}^{P}}{\alpha_{\mathrm{CO}_{2}}^{\mathrm{RBC}}}\left( 1-h\left( r \right) \right)u_{P}\left( r \right)+h\left( r \right)u_{\mathrm{RBC}}\left( r \right)+h\left( r \right)u_{\mathrm{RBC}}\left( r \right)\frac{\partial C_{\mathrm{HbCO}_{2,T}}}{\partial C_{\mathrm{CO}_{2}}^{\mathrm{RBC}}}+h\left( r \right)u_{\mathrm{RBC}}\left( r \right)K^{',RBC}f_{\mathrm{water}}\frac{\beta_{\mathrm{RBC}}}{C_{H^{+}}^{\mathrm{RBC}}(2.303C_{\mathrm{HCO}_{3}^{-}}^{\mathrm{RBC}}+\beta_{\mathrm{RBC}})} \right]\frac{\partial C_{\mathrm{CO}_{2}}^{\mathrm{RBC}}}{\partial z}=\left( \frac{\alpha_{\mathrm{CO}_{2}}^{P}}{\alpha_{\mathrm{CO}_{2}}^{\mathrm{RBC}}} \right)\frac{D_{\mathrm{CO}_{2}}^{P}}{r}\frac{\partial}{\partial r}\left( r\frac{\partial C_{\mathrm{CO}_{2}}^{\mathrm{RBC}}}{\partial r} \right)-\left( 1-h\left( r \right) \right)R_{\mathrm{HCO}_{3}^{-}}^{P}+h\left( r \right)J_{\mathrm{HCO}_{3}^{-}}\left[ \frac{s}{v} \right]_{\mathrm{RBC}}.$ | **A.21** |
| --- | --- | --- |

Equation **7** can be obtained from Eq. **A.21** by inserting $C_{\mathrm{CO}_{2}}^{\mathrm{RBC}}=\alpha_{\mathrm{CO}_{2}}^{\mathrm{RBC}}P_{CO_{2}}$.

Inside RBCs, carbon dioxide binds to the α- and β-chains of hemoglobin. Following (Kilmartin and Rossi-Bernardi, 1973), the total CO_2_ bound to hemoglobin in the presence of 2,3-Diphosphoglycerate (DPG), a RBC metabolic intermediate, can be described as

|  | $C_{\mathrm{HbCO}_{2,T}}=\frac{C_{\mathrm{HbT}}}{4}\left[ \frac{2\lambda_{\alpha}C_{\mathrm{CO}_{2}}^{\mathrm{RBC}}}{1+\lambda_{\alpha}C_{\mathrm{CO}_{2}}^{\mathrm{RBC}}}+\frac{2\lambda_{\beta}C_{\mathrm{CO}_{2}}^{\mathrm{RBC}}}{1+\lambda_{\beta}C_{\mathrm{CO}_{2}}^{\mathrm{RBC}}+K_{\mathrm{DPG}}C_{\mathrm{DPG}}} \right],$ | **A.22** |
| --- | --- | --- |

where $\lambda_{\alpha}$ and $\lambda_{\beta}$ are the association constant for CO_2_ binding to the α- and β-chains of hemoglobin, respectively, $K_{\mathrm{DPG}}$ and $C_{\mathrm{DPG}}$ are the association constant for DPG and hemoglobin and the concentration of DPG, respectively. The derivative of the total hemoglobin carbamate with respect to RBC carbon dioxide concentration in Eqs. **A.21** and **7** is then given as

|  | $\frac{\partial C_{\mathrm{HbCO}_{2,T}}}{\partial C_{\mathrm{CO}_{2}}^{\mathrm{RBC}}}=\frac{C_{\mathrm{HbT}}}{4}\left[ \frac{2\lambda_{a}}{{(1+\lambda_{a}C_{\mathrm{CO}_{2}}^{\mathrm{RBC}})}^{2}}+\frac{2\lambda_{b}(1+K_{\mathrm{DPG}}C_{\mathrm{DPG}})}{{(1+\lambda_{b}C_{\mathrm{CO}_{2}}^{\mathrm{RBC}}+K_{\mathrm{DPG}}C_{\mathrm{DPG}})}^{2}} \right].$ | **A.23** |
| --- | --- | --- |

The anion transporter exchanges bicarbonate and chloride ions between plasma and RBC. Following Huang and Hellums (Huang, 1991; Huang and Hellums, 1994), the flux over the RBC membrane is defined (positive for influx of bicarbonate into RBC and outflux of chloride from the RBC) as

|  | $J_{\mathrm{HCO}_{3}^{-}}=C_{\mathrm{AT}}k_{\mathrm{trans}}K_{A}\left( C_{\mathrm{Cl}^{-}}^{\mathrm{RBC}}C_{\mathrm{HCO}_{3}^{-}}^{P}-C_{\mathrm{Cl}^{-}}^{P}C_{\mathrm{HCO}_{3}^{-}}^{\mathrm{RBC}} \right)/$  $\left[ {(C}_{\mathrm{Cl}^{-}}^{\mathrm{RBC}}+C_{\mathrm{Cl}^{-}}^{P}+C_{\mathrm{HCO}_{3}^{-}}^{\mathrm{RBC}}+C_{\mathrm{HCO}_{3}^{-}}^{P} \right)$  $+2K_{A}(C_{\mathrm{Cl}^{-}}^{\mathrm{RBC}}C_{\mathrm{Cl}^{-}}^{P}+C_{\mathrm{HCO}_{3}^{-}}^{\mathrm{RBC}}C_{\mathrm{HCO}_{3}^{-}}^{P}+C_{\mathrm{Cl}^{-}}^{\mathrm{RBC}}C_{\mathrm{HCO}_{3}^{-}}^{P}+C_{\mathrm{Cl}^{-}}^{P}C_{\mathrm{HCO}_{3}^{-}}^{\mathrm{RBC}})]$ | **A.24** |
| --- | --- | --- |

where $k_{\mathrm{trans}}$ and $K_{A}$ are the translocation and the equilibrium association rate constants, respectively, and $C_{\mathrm{AT}}$ is the anion transporter surface concentration defined as $C_{\mathrm{AT}}=T_{\mathrm{tot}}/(N_{A}s_{\mathrm{RBC}})$, where $T_{\mathrm{tot}}$, $N_{A}$ and $s_{\mathrm{RBC}}$ are the total number of transporter molecules per RBC, the Avogadro number and the surface area of an RBC, respectively.

# Calculation of reported values

Calculation of reported values regarding oxygen transport were presented in (Olgac and Kurtcuoglu, 2015a). Here we present the calculation of reported values regarding carbon dioxide transport. Concentration of any species in plasma and RBC at the vessel outlet of each order is calculated as a plasma and RBC volumetric flow-weighted average:

|  | $C_{outlet,i}^{\mathrm{RBC}}= \frac{\int_{0}^{R} \left[ h(r)u_{\mathrm{RBC}}\left( r \right)C^{\mathrm{RBC}} \right]2\pi rdr}{\int_{0}^{R} \left[ h(r)u_{\mathrm{RBC}}\left( r \right) \right]2\pi rdr},$ | **A.25**(a) |
| --- | --- | --- |
|  | $C_{outlet,i}^{p}= \frac{\int_{0}^{R} \left[ \left( 1-h\left( r \right) \right)u_{P}\left( r \right)C^{P} \right]2\pi rdr}{\int_{0}^{R} \left[ \left( 1-h\left( r \right) \right)u_{P}\left( r \right) \right]2\pi rdr}.$ | **A.25**(b) |

Equation A.25 is also used to calculate pH_RBC_ and pH_P_ at the outlet of each Strahler order by replacing $C^{\mathrm{RBC}}$ and $C^{P}$ with pH_RBC_ and pH_P_, respectively. Similarly, the partial pressure of oxygen at the vessel outlet of each order is calculated as

|  | $P_{\mathrm{CO}_{2},outlet,i}= \frac{\int_{0}^{R} \left[ \left( 1-h\left( r \right) \right)u_{P}\left( r \right)P_{CO_{2}} \right]2\pi rdr}{\int_{0}^{R} \left[ \left( 1-h\left( r \right) \right)u_{P}\left( r \right) \right]2\pi rdr}.$ | **A.26** |
| --- | --- | --- |

The cortical tissue $P_{\mathrm{CO}_{2}}$ is calculated as a volume-weighted average:

|  | $P_{\mathrm{CO}_{2},C}=\frac{\sum_{j} P_{CO_{2},j}V_{j}}{\sum_{j} V_{j}} ,$ | **A.27** |
| --- | --- | --- |

where *j* is each individual computational cell that lies in the cortical tissue and $V_{j}$ is the volume of that cell.

Oxygen delivery, $D_{O_{2}}$, and carbon dioxide delivery excluding plasma bicarbonate, $D_{\mathrm{CO}_{2}}$, at any position along the vasculature are calculated as

|  | $D_{O_{2}}= \int_{0}^{R} \left[ \begin{aligned} h\left( r \right)u_{\mathrm{RBC}}\left( r \right)C_{\mathrm{HbO}_{2}}^{\mathrm{RBC}}+h\left( r \right)u_{\mathrm{RBC}}\left( r \right)C_{O_{2}}^{\mathrm{RBC}} \\ +\left( 1-h\left( r \right) \right)u_{P}\left( r \right)C_{O_{2}}^{P} \end{aligned} \right]2\pi rdr$, | **A.28**(a) |
| --- | --- | --- |
|  | $D_{\mathrm{CO}_{2}}= \int_{0}^{R} \left[ \begin{aligned} h\left( r \right)u_{\mathrm{RBC}}\left( r \right)C_{\mathrm{HbCO}_{2,T}}+h\left( r \right)u_{\mathrm{RBC}}\left( r \right)C_{CO_{2}}^{\mathrm{RBC}} \\ +{h\left( r \right)u_{\mathrm{RBC}}\left( r \right)C}_{\mathrm{HCO}_{3}^{-}}^{\mathrm{RBC}} \\ +\left( 1-h\left( r \right) \right)u_{P}\left( r \right)C_{\mathrm{CO}_{2}}^{P} \end{aligned} \right]2\pi rdr,$ | **A.28**(b) |

where R is the vessel radius at that location. Plasma and RBC oxygen concentrations in the above equations are determined as $C_{O_{2}}^{P}=\alpha_{O_{2}}^{P}P_{O_{2}}$ and $C_{O_{2}}^{\mathrm{RBC}}=\alpha_{O_{2}}^{\mathrm{RBC}}P_{O_{2}}$, respectively. Similarly, plasma and RBC carbon dioxide concentrations are determined as $C_{\mathrm{CO}_{2}}^{P}=\alpha_{\mathrm{CO}_{2}}^{P}P_{CO_{2}}$ and $C_{\mathrm{CO}_{2}}^{\mathrm{RBC}}=\alpha_{\mathrm{CO}_{2}}^{\mathrm{RBC}}P_{\mathrm{CO}_{2}}$, respectively, whereas hemoglobin-bound carbon dioxide concentration in RBCs is determined through Eq. **A.22** as detailed above. This carbon dioxide delivery given by Eq. **A.28** refers to the sum of deliveries of all forms of CO_2_, except $\mathrm{HCO}_{3}^{-}$ in plasma, whereas the total carbon dioxide delivery refers to the sum of deliveries of all forms of CO_2_ defined as:

|  | $D_{\mathrm{CO}_{2},T}= \int_{0}^{R} \left[ \begin{aligned} h\left( r \right)u_{\mathrm{RBC}}\left( r \right)C_{\mathrm{HbCO}_{2,T}} \\ +h\left( r \right)u_{\mathrm{RBC}}\left( r \right)C_{CO_{2}}^{\mathrm{RBC}}+\left( 1-h\left( r \right) \right)u_{P}\left( r \right)C_{\mathrm{CO}_{2}}^{P} \\ +{h\left( r \right)u_{\mathrm{RBC}}\left( r \right)C}_{\mathrm{HCO}_{3}^{-}}^{\mathrm{RBC}}+\left( 1-h\left( r \right) \right)u_{P}\left( r \right)C_{\mathrm{HCO}_{3}^{-}}^{P} \end{aligned} \right]2\pi rdr$. | **A.29** |
| --- | --- | --- |

The delivery of other species in plasma and in RBC at any position along the vasculature are calculated, respectively, as

|  | $D_{\mathrm{Cl}_{\mathrm{RBC}}^{-}}= \int_{0}^{R} \left[ h\left( r \right)u_{\mathrm{RBC}}\left( r \right)C_{\mathrm{Cl}^{-}}^{\mathrm{RBC}} \right]2\pi rdr$, | **A.30**(a) |
| --- | --- | --- |
|  | $D_{H_{P}^{+}}= \int_{0}^{R} \left[ \left( 1-h\left( r \right) \right)u_{P}\left( r \right)C_{H^{+}}^{P} \right]2\pi rdr$, | **A.30**(b) |
|  | $D_{\mathrm{HCO}_{3,P}^{-}}= \int_{0}^{R} \left[ \left( 1-h\left( r \right) \right)u_{P}\left( r \right)C_{\mathrm{HCO}_{3}^{-}}^{P} \right]2\pi rdr$, | **A.30**(c) |
|  | $D_{\mathrm{Cl}_{P}^{-}}= \int_{0}^{R} \left[ \left( 1-h\left( r \right) \right)u_{P}\left( r \right)C_{\mathrm{Cl}^{-}}^{P} \right]2\pi rdr$. | **A.30**(d) |

The total arterial carbon dioxide and venous oxygen fluxes are calculated as

|  | ${J_{\mathrm{CO}_{2},a}=D}_{CO_{2},T,inlet,a,10}-D_{\mathrm{CO}_{2},T,outlet,a,0},$ | **A.31**(a) |
| --- | --- | --- |
|  | ${J_{O_{2},v}=D}_{O_{2},inlet,v,0}-D_{O_{2},outlet,v,10}$. | **A.31**(b) |

In the above equations, positive flux represents flux from the vessel into the tissue, whereas negative flux denotes the opposite, i.e., negative $J_{O_{2},v}$ and $J_{\mathrm{CO}_{2},a}$ is oxygen shunted to the venous tree and carbon dioxide shunted to the arterial tree, respectively.

# Venous return boundary conditions

Here we detail boundary conditions 1 & 2 on venous return and how they are implemented.

## Condition 1: Balanced CO_2_ distribution on the venous return

This condition assumes chemical equilibrium both in RBCs and plasma ($R_{\mathrm{HCO}_{3}^{-},inlet,v,0}^{P}=0$) and zero flux of bicarbonate and chloride ions over the RBC membrane ($J_{\mathrm{HCO}_{3}^{-},inlet, v,0}=0$). It is also assumed that the total CO_2_ production is distributed into all forms of CO_2_ on venous return, i.e., CO_2_ in plasma and RBC, $\mathrm{HCO}_{3}^{-}$ in plasma and RBC, and hemoglobin bound CO_2_. The venous return $P_{\mathrm{CO}_{2},inlet,v,0}$, $C_{H^{+},inlet,v,0}^{\mathrm{RBC}}$, $C_{\mathrm{HCO}_{3}^{-},inlet,v,o}^{\mathrm{RBC}}$ and $C_{\mathrm{HCO}_{3}^{-},inlet,v,o}^{P}$ are specified such that

|  | $D_{\mathrm{CO}_{2},T,inlet,v,0}=D_{CO_{2},T, outlet,a,0}+\dot{V}_{\mathrm{CO}_{2},M}+\dot{V}_{CO_{2},C},$ | **A.32** |
| --- | --- | --- |
|  | $J_{\mathrm{HCO}_{3}^{-}, inlet, v,0}=0,$ |  |

where $\dot{V}_{O_{2},M}$ and $\dot{V}_{O_{2},C}$ are the medullary and cortical carbon dioxide production rate, respectively, and $D_{\mathrm{CO}_{2},T}$ is the total delivery of carbon dioxide in all forms (see above for calculation). In assuring the latter in Eq. **A.32**, $C_{\mathrm{HCO}_{3}^{-},inlet,v,o}^{P}$ is specified as

|  | $C_{\mathrm{HCO}_{3}^{-},inlet,v,o}^{P}=\frac{C_{\mathrm{Cl}^{-},inlet,v,o}^{P}}{C_{\mathrm{Cl}^{-},inlet,v,o}^{\mathrm{RBC}}}C_{\mathrm{HCO}_{3}^{-},inlet,v,o}^{\mathrm{RBC}},$ | **A.33** |
| --- | --- | --- |

for which the plasma – RBC chloride concentration ratio is assumed to be equal to that in the outlet of the afferent arteriole

|  | $\frac{C_{\mathrm{Cl}^{-},inlet,v,o}^{P}}{C_{\mathrm{Cl}^{-},inlet,v,o}^{\mathrm{RBC}}}=\frac{C_{\mathrm{Cl}^{-},outlet,a,o}^{P}}{C_{\mathrm{Cl}^{-},outlet,a,o}^{\mathrm{RBC}}},$ | **A.34** |
| --- | --- | --- |

where $C_{\mathrm{Cl}^{-},outlet,a,o}^{P}$ and $C_{\mathrm{Cl}^{-},outlet,a,o}^{\mathrm{RBC}}$ are calculated with Eq. **A.25**. Equation **A.34** is used to conserve the total chloride delivery on venous return such that

|  | $D_{\mathrm{Cl}_{P}^{-}, inlet,v,0}+D_{\mathrm{Cl}_{\mathrm{RBC}}^{-}, inlet,v,0}=D_{\mathrm{Cl}_{P}^{-}, outlet,a,0}+D_{\mathrm{Cl}_{\mathrm{RBC}}^{-}, outlet,a,0}$. | **A.35** |
| --- | --- | --- |

The last species to set on the venous return, i.e., $C_{H^{+}}^{P}$, is specified such that $R_{\mathrm{HCO}_{3}^{-},inlet,v,0}^{P}=0$:

|  | ${C_{H^{+},inlet,v,0}^{P}=K}_{\mathrm{RBC}}^{'}\alpha_{\mathrm{CO}_{2}}^{P}\frac{P_{CO_{2}}}{C_{\mathrm{HCO}_{3}^{-}}^{P}}.$ | **A.36** |
| --- | --- | --- |

## Condition 2: Unbalanced CO_2_ distribution on the venous return

This condition assumes chemical equilibrium both in RBCs and plasma ($R_{\mathrm{HCO}_{3}^{-},inlet,v,0}^{P}=0$), but does not impose zero flux of bicarbonate and chloride ions over the RBC membrane, i.e., $J_{\mathrm{HCO}_{3}^{-},inlet, v,0}\neq0$. It is also assumed that the total CO_2_ production is distributed into all forms of CO_2_ on venous return except $\mathrm{HCO}_{3}^{-}$ in plasma. The venous return $P_{\mathrm{CO}_{2},inlet,v,0}$, $C_{H^{+},inlet,v,0}^{\mathrm{RBC}}$ and $C_{\mathrm{HCO}_{3}^{-},inlet,v,o}^{\mathrm{RBC}}$ are specified such that

|  | $D_{\mathrm{CO}_{2},inlet,v,0}=D_{CO_{2}, outlet,a,0}+\dot{V}_{\mathrm{CO}_{2},M}+\dot{V}_{\mathrm{CO}_{2},C},$ | **A.37** |
| --- | --- | --- |

where $D_{\mathrm{CO}_{2}}$ is the delivery of carbon dioxide excluding plasma bicarbonate (see above for calculation). Plasma bicarbonate delivery is conserved as

|  | $D_{\mathrm{HCO}_{3,P}^{-},inlet,v,0}=D_{\mathrm{HCO}_{3,P}^{-},outlet,a,0},$ | **A.38** |
| --- | --- | --- |

whereas chloride ion delivery is conserved as in Condition 1 with Eqs. **A.34** and **A.35**, and hydrogen ion in plasma, $C_{H^{+}}^{P}$, is specified such that $R_{\mathrm{HCO}_{3}^{-},inlet,v,0}^{P}=0$ with Eq. **A.36**.

In both conditions 1 & 2, the BCs ensure that the total carbon dioxide delivery throughout the kidney is conserved, i.e., $D_{CO_{2},T,inlet,a,10}=D_{\mathrm{CO}_{2},T,outlet,v,10}-\dot{V}_{\mathrm{CO}_{2},C}-\dot{V}_{\mathrm{CO}_{2},M}$.

# Grid independence study

Five sets of non-uniform unstructured grids, respectively consisting of 570,726 (*coarsest*) 1,352,832 (*coarse*), 2,642,250 (*medium*), 4,565,808 (*fine*) and 7,726,664 (*finest*) hexahedral cells, were generated to cover the computational domain and evaluated for the solution of Eqs. **7 - 18**. Comparisons were made between the converged steady-state results obtained with two consecutively finer grids for the base case. The relative error between the subsequent grids is defined as (Olgac and Kurtcuoglu, 2015a)

|  | $e_{finer-coarser}=\left\vert\frac{\varepsilon_{\mathrm{finer}}\left( x \right)-\varepsilon_{\mathrm{coarser}}\left( x \right)}{\varepsilon_{\mathrm{finer}}\left( x \right)} \right\vert,$ | **A.39** |
| --- | --- | --- |

where $e$ is the relative error, $\varepsilon$ is the particular result being compared, $x$ is the spatial location, “finer” refers to grid with a larger number of cells, and “coarser” to the one with the smaller number. All relative errors between subsequently finer grids are presented in Table 1 below. The fine grid with 4,565,808 elements was sufficient to accurately calculate $P_{\mathrm{CO}_{2}}$ and $J_{\mathrm{CO}_{2},a}$ within a relative error margin of 0.2 % and 3.2 %, respectively, compared with the finest grid. Hence, the fine grid was utilized in all computations performed in this study.

**Table 1.** Relative errors (%) in $P_{\mathrm{CO}_{2}}$ and $J_{\mathrm{CO}_{2},a}$ between subsequently finer grids.

|  | $P_{\mathrm{CO}_{2},a}$ | $P_{CO_{2},v}$ | $P_{\mathrm{CO}_{2},T}$ | $J_{\mathrm{CO}_{2},a}$ |
| --- | --- | --- | --- | --- |
| $e_{coarse-coarsest}$ | 1.3 | 1.7 | 1.5 | 21.0 |
| $e_{medium-coarse}$ | 1.3 | 1.6 | 1.4 | 23.4 |
| $e_{fine-medium}$ | 0.4 | 0.4 | 0.4 | 6.2 |
| $e_{finest-fine}$ | 0.1 | 0.2 | 0.2 | 3.2 |

$P_{\mathrm{CO}_{2}}$, partial pressure of carbon dioxide (arterial $(P_{\mathrm{CO}_{2},a}$), venous $(P_{\mathrm{CO}_{2},v}$) and tissue $(P_{\mathrm{CO}_{2},T}$)); $J_{\mathrm{CO}_{2},a}$, flux of carbon dioxide between the arteries and the tissue. The reported relative errors for $P_{CO_{2}}$ are the maximum amongst all Strahler orders, whereas for $J_{\mathrm{CO}_{2},a}$, maximum relative error amongst orders 0 – 5 are reported, since $J_{CO_{2}}$ assumes close-to-zero values for higher orders.
