# Supplementary figures and images for: The Bohr Effect Is Not a Likely Promoter of Renal Preglomerular Oxygen Shunting
Source: Front Physiol. 2016 Oct 27;7:482. doi: 10.3389/fphys.2016.00482 (PMC5081373; doi:10.3389/fphys.2016.00482)

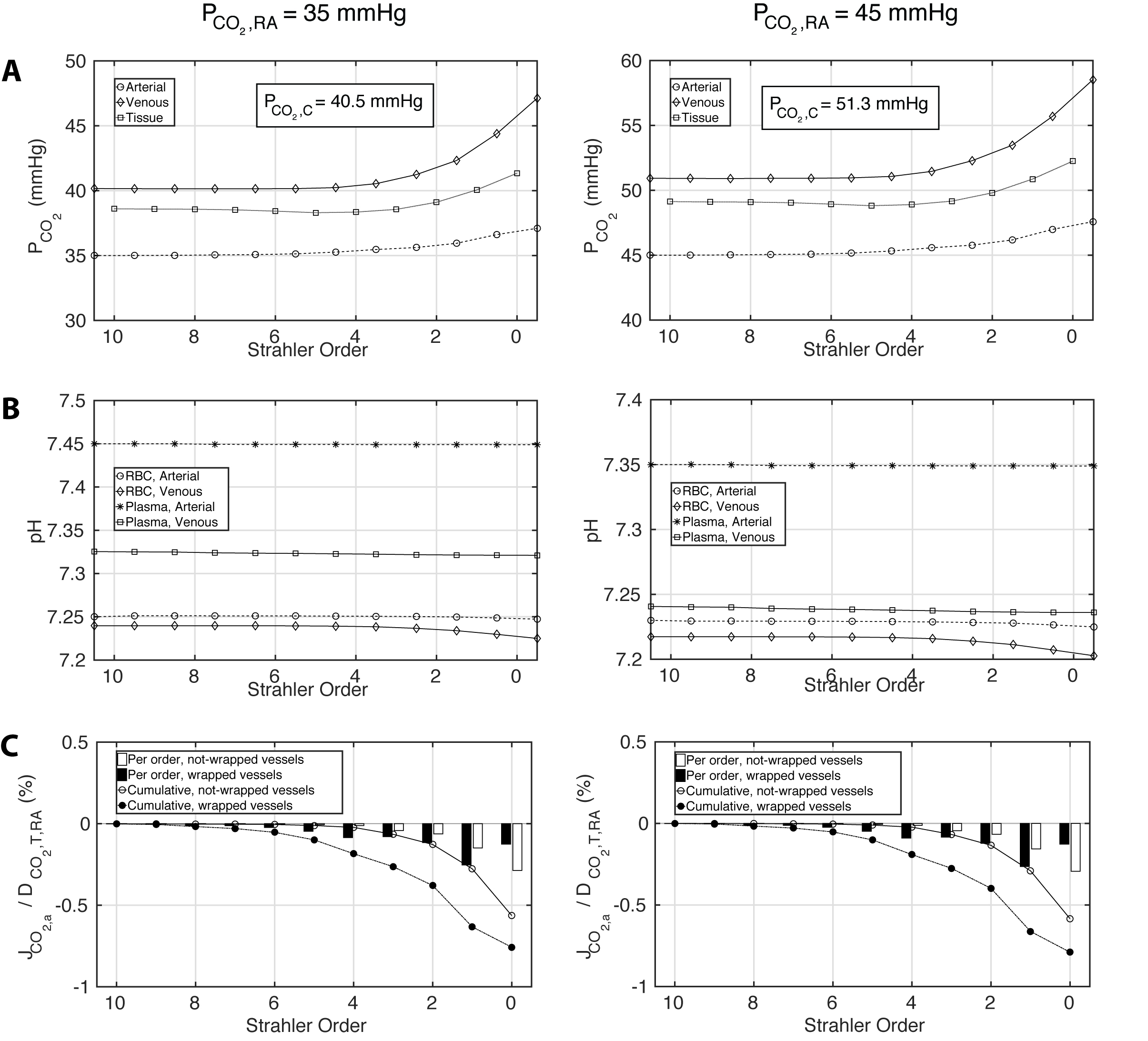

Supplement: Supplementary file 6 [file Image1.JPEG]

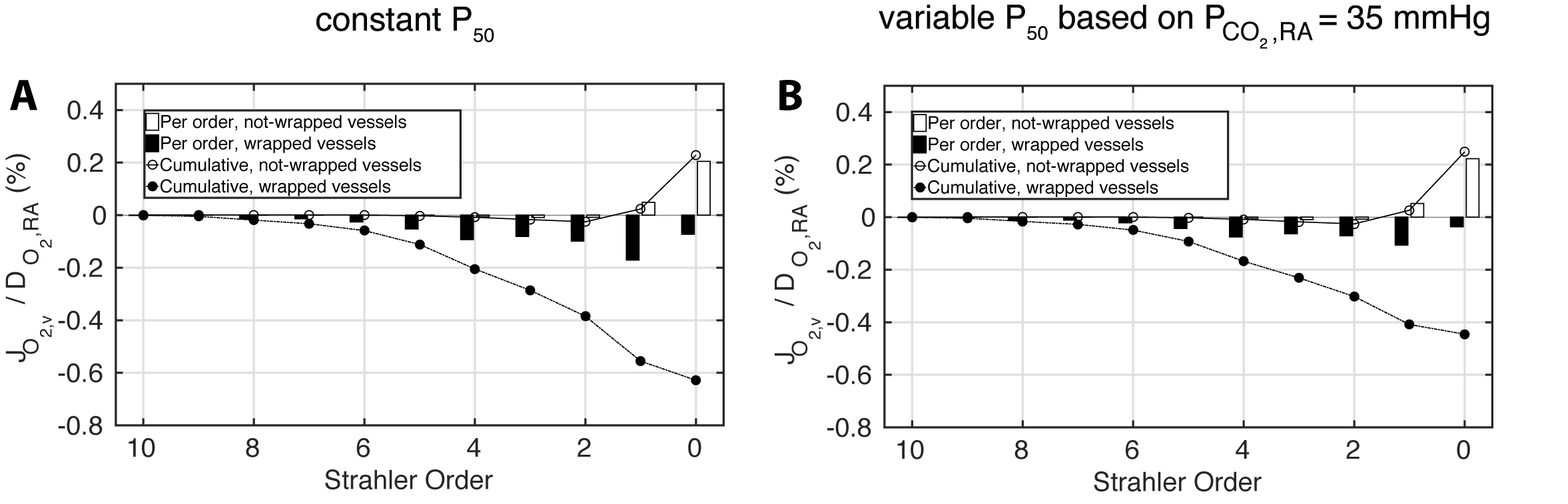

Supplement: Supplementary file 7 [file Image2.JPEG]

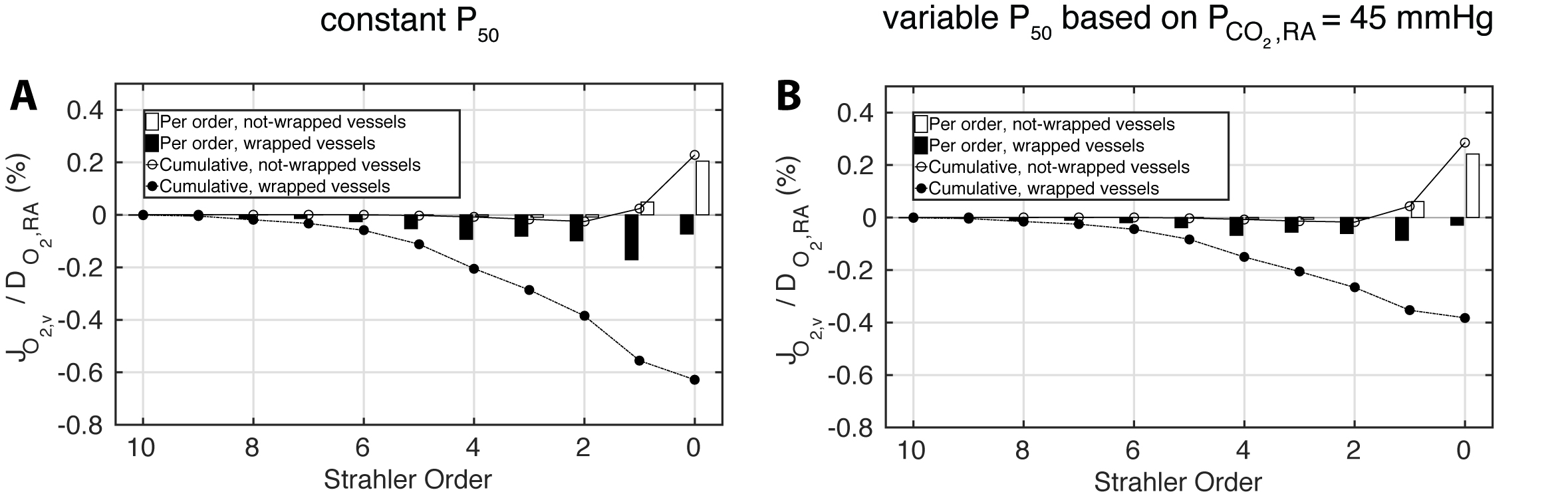

Supplement: Supplementary file 8 [file Image3.JPEG]

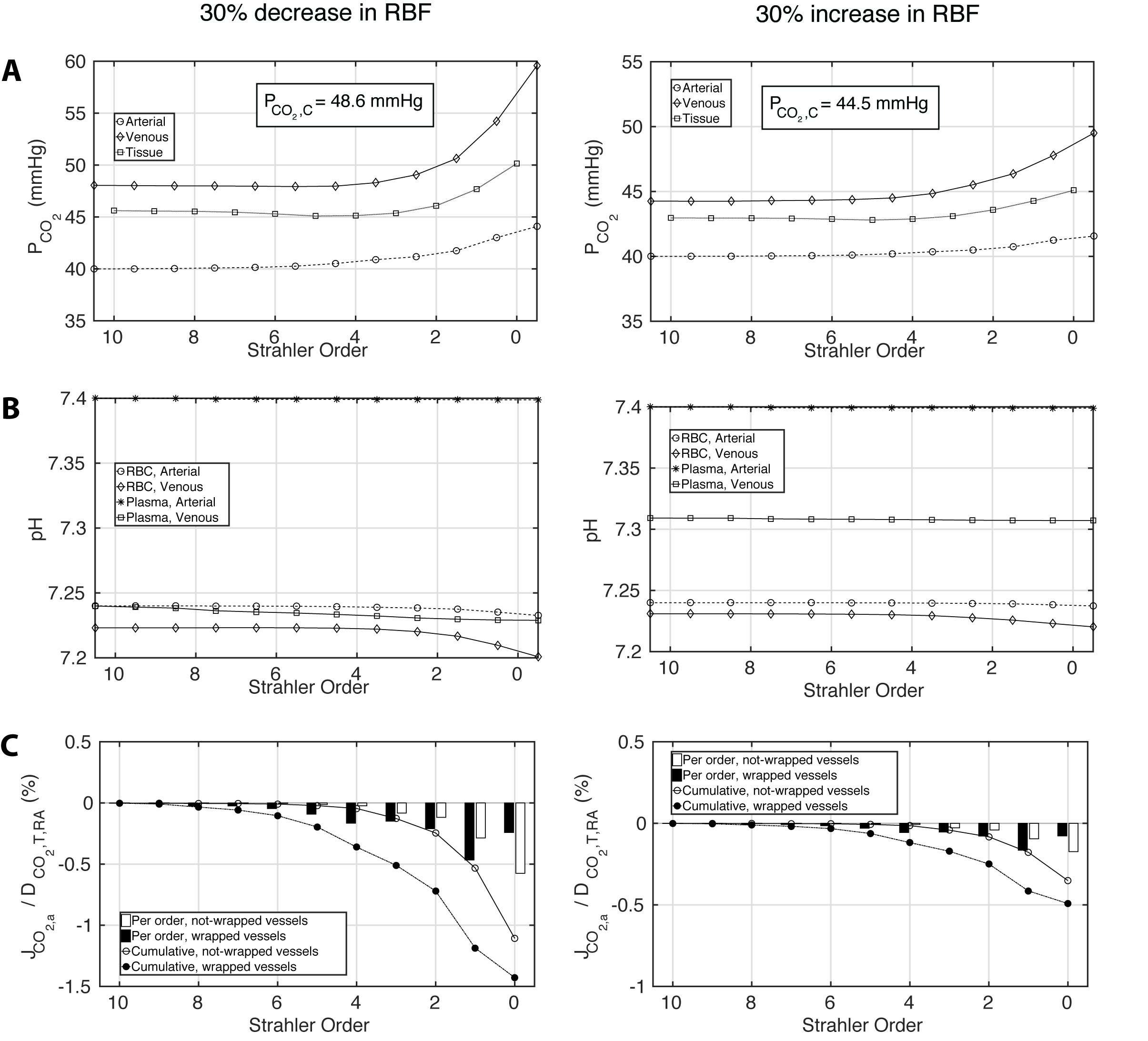

Supplement: Supplementary file 9 [file Image4.JPEG]

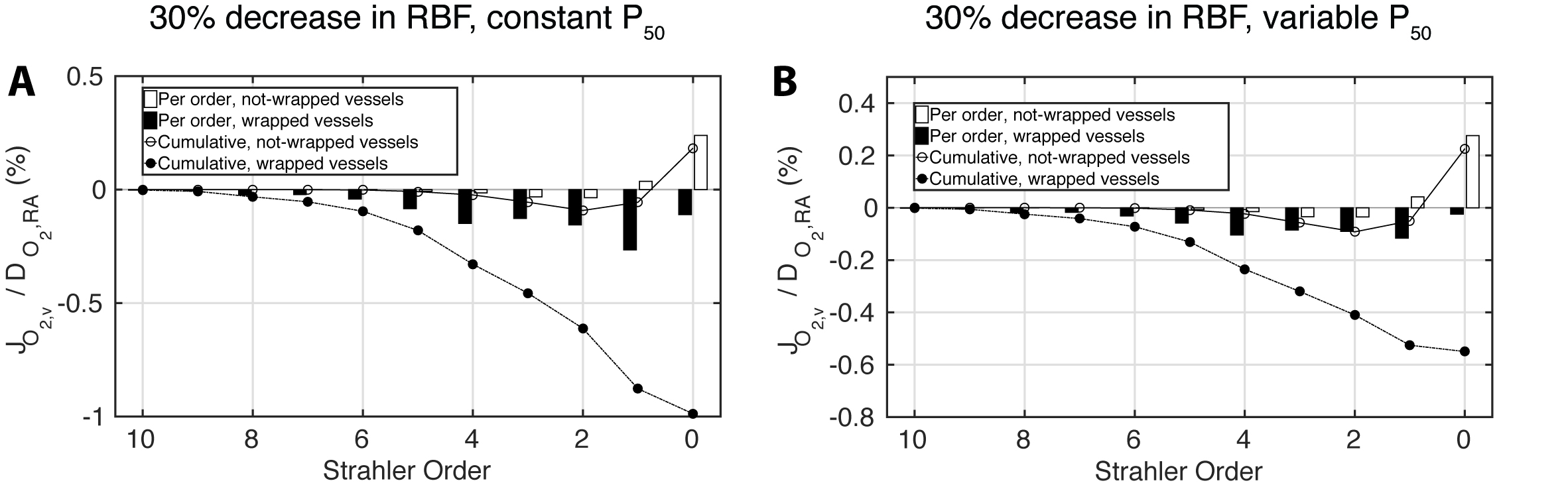

Supplement: Supplementary file 10 [file Image5.JPEG]

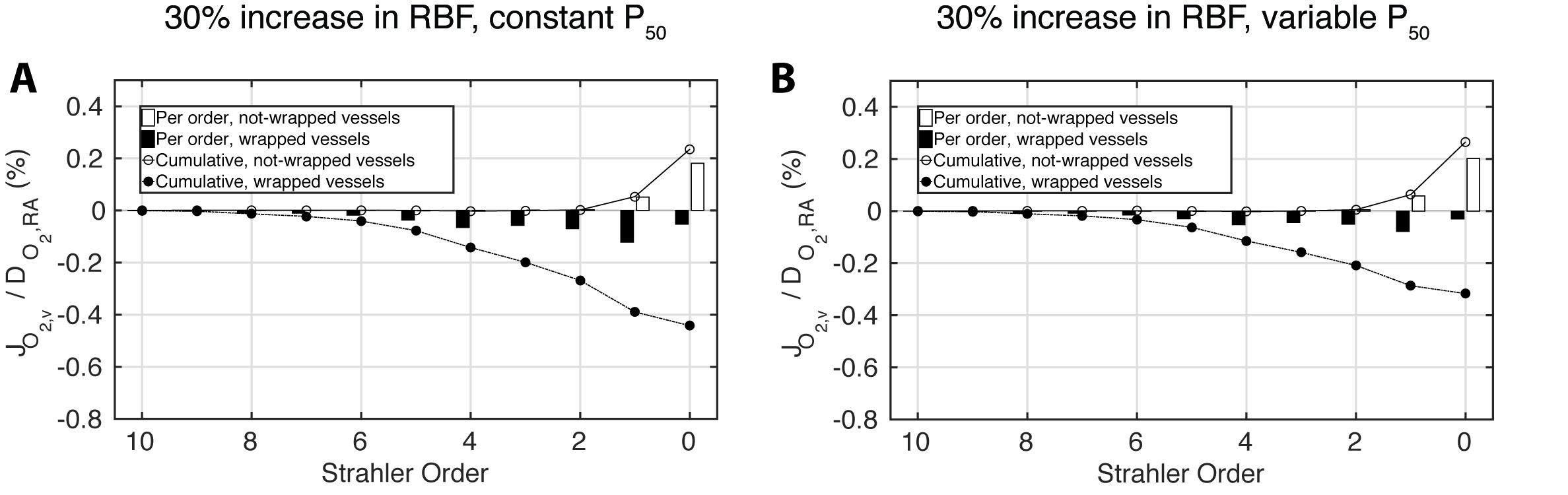

Supplement: Supplementary file 11 [file Image6.JPEG]
